# Botulinum Neurotoxin-A Injected Intrastriatally into Hemiparkinsonian Rats Improves the Initiation Time for Left and Right Forelimbs in Both Forehand and Backhand Directions

**DOI:** 10.3390/ijms20040992

**Published:** 2019-02-25

**Authors:** Veronica Antipova, Carsten Holzmann, Alexander Hawlitschka, Andreas Wree

**Affiliations:** 1 Institute of Anatomy, Rostock University Medical Center, D-18057 Rostock, Germany; veronica.antipova@medunigraz.at (V.A.); alexander.hawlitschka@med.uni-rostock.de (A.H.); 2 Gottfried Schatz Research Center for Cell Signaling, Metabolism and Aging, Macroscopic and Clinical Anatomy, Medical University of Graz, A-8010 Graz, Austria; 3 Institute of Medical Genetics, Rostock University Medical Center, D-18057 Rostock, Germany; carsten.holzmann@med.uni-rostock.de; 4 Centre of Transdisciplinary Neuroscience Rostock, D-18147 Rostock, Germany

**Keywords:** botulinum neurotoxin-A, stepping test, initiation time, hemiparkinsonian rat, 6-OHDA, basal ganglia, striatum, correlation analysis

## Abstract

Forelimb stepping is a widely used test for the assessment of forelimb akinesia in hemiparkinsonian (hemi-PD) rats. The initiation time (IT) is considered the most sensitive parameter in the stepping test procedure. Here we propose a novel, reliable, and simple method for the measurement of IT of both forelimbs in both forehand and backhand directions in rats. Evaluating the same videos taken for quantifying adjusting steps, IT measurements were done without additional experiments. This is in contrast to the classical approach introduced by Olsson et al. (1995), in which separate experiments are necessary. We successfully applied our approach to hemi-PD rats intrastriatally treated with botulinum neurotoxin-A (BoNT-A). In naïve rats, an IT of about 0.62 s was found, and in right-sided hemi-PD rats the IT of the left forepaw increased to about 3.62 s. These hemi-PD rats showed, however, reduced ITs of the impaired left forepaws 1 month and the second time 7 months after induction of hemi-PD via the injection of 1 ng BoNT-A into the ipsilateral striatum, depending on post BoNT-A survival time. The method described offers the possibility of a precise and animal-friendly evaluation of IT in rats, including the beneficial effect of BoNT-A treatment in hemi-PD rats.

## 1. Introduction

In Parkinson’s disease (PD), bradykinesia and postural and gait impairments are leading symptoms [1,2] that are based on delayed movement initiation [3,4,5,6]. The 6-hydroxydopamine (6-OHDA)-induced hemiparkinsonian (hemi-PD) rat model is well characterized with respect to behavioral, biochemical, and morphological outcome [7,8,9,10,11,12,13,14,15]. The catecholamine selective neurotoxin 6-OHDA has been widely used as a tool to produce dopamine (DA) axon terminal lesions in rats by injection into the medial forebrain bundle (MFB), the substantia nigra pars compacta (SNpc), or in various parts of the caudate-putamen (CPu = striatum) to induce a rat hemi-PD model [16,17,18,19].

The extent of the induced motor deficits depends on the site and dosage of 6-OHDA injected. An injection of 6-OHDA (10–24 µg) into the MFB produces profound neurodegeneration of the tyrosine hydroxylase-immunoreactive (TH-ir) neurons in the ipsilateral SNpc, followed by striatal DA depletion and significant motor deficits, mimicking a late stage of PD [20,21,22].

For behavioral characterization of hemi-PD or to measure the movement impairments of rats, drug-induced or spontaneous motor tests are used [5,23,24,25,26].

With respect to spontaneous motor behavior, in 1995 Olsson et al. [5] showed marked and long-lasting impairments of the number of adjusting steps (AS) of the forepaw contralateral to the 6-OHDA-lesioned hemisphere, and also deficits of the initiation time (IT) as defined by that group in the respective forelimb [5,27,28]. The impairments of movement initiation are comparable with the deficits in the initiation of movement present in PD patients [1,29,30]. The IT in particular is considered an index for motor initiation deficits and akinesia, and generally believed the most sensitive parameter in the stepping test procedure [1,3,25,31,32].

The currently and widely performed method determining the IT of stepping was proposed by Olsson et al. [5] in an experiment separate from monitoring the AS. In brief: during the first 3 days the rats were handled by the experimenter to familiarize them with the experimenter’s grip and test procedure. A wooden ramp 1.1 m long connecting a wooden table with the rat´s home cage is used. In 2 days rats had to learn to run spontaneously up the ramp into their home cage.

In the test done by Olsson et al. [5], the experimenter holds the hind part of the rat’s body with one hand slightly above the surface, and with the other hand fixes the forelimb not being monitored. IT is measured from the moment the experimenter puts the rat´s free forelimb on the base of the ramp to the time point the rat initiated movement with that forelimb. Using this procedure, the IT in naïve rats is in the range of about 1–2 s [5]. Contralateral to an MFB lesion, the IT is significantly prolonged to about 60–120 s [5]. However, the published values of ITs of other researchers using the Olsson method differed considerably: between 10 s and 20 s in the hampered forelimb [30,31,33,34,35,36,37]. The IT of the ipsilateral unaffected forelimb mostly lies in the range of the values of naïve rats. According to Olsson et al. [5], for each rat one IT is measured for the right and one for the left forelimb. After handling (3 days) and learning to run the ramp (2 days), testing in each rat consists of two tests per day for 3 consecutive days. Subsequently, the mean of the six tests is calculated. Thus, the overall IT evaluation in rats as per Olsson et al. [5] takes 8 days. Supplemental Tables S1 and S2 summarize results of previous 6-OHDA experiments evaluating AS and, in about one third of them, also IT measured according to Olsson et al. [5].

In the present study, we propose a new, reliable and simple method which allows the analysis of the IT in videos taken originally for the measurement of the number of AS for left and right forepaws, in both the forehand and backhand directions [38]. In contrast to Olsson et al. [5], a further time-consuming experiment to study the IT is not necessary. In expansion of the method of Olsson et al. [5], the present approach allows access to the IT of stepping of the left and right forepaws not only in one direction, i.e., ”in the direction of the home cage”. Our approach evaluates the IT of forelimb use from videos monitoring the AS of left and right forelimbs both in forehand and also in backhand directions. In each rat, testing consists of two stepping tests per day for three consecutive days, and the four values (right forelimb in forehand and backhand directions; left forelimb in forehand and backhand directions) are calculated as means of the respective six tests. Importantly, the method needs no additional equipment or additional adaptation to an experimental device and procedure as compared with Olsson et al. [5]. Clearly speaking, the IT presented here is a “side product” of the videos taken for evaluating the number of AS, refining the characterization of the animal´s behavior and reducing animal experiments.

We applied our new approach for the determination of the IT in the model of hemi-PD rats which were intrastriatally treated with botulinum neurotoxin-A (BoNT-A) [38,39,40,41,42,43,44,45]. The hypothesis tested is whether intrastriatal BoNT-A could improve the initiation time of stepping in hemi-PD rats.

In PD, DA depletion leads to hyperactivity of cholinergic interneurons in the CPu [46,47,48,49] which likely contributes to major motor symptoms [50,51]. BoNT-A is thought to act as a local anticholinergic drug [38,39,40,41,42,43,44,45]. As shown, unilateral injection of 1 ng BoNT-A into the CPu of hemi-PD rats temporarily reduced apomorphine-induced rotation behavior significantly for at least 3 months [38,39,40,41,42,43,44,45]. As shown in various studies, 1 ng BoNT-A intrastriatally injected into hemi-PD rats is a well-tolerated and a therapeutically adequate dose (reviewed in [43]).

**Figure 1:**
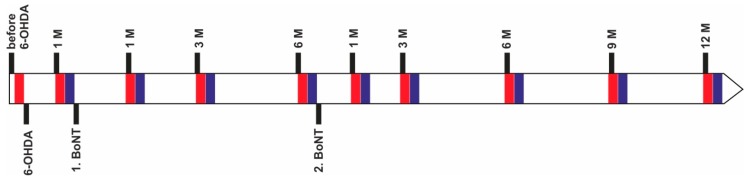
Time scale of 6-hydroxydopamine (6-OHDA) lesion and behavioral tests in rats after the first (1. botulinum neurotoxin (BoNT)) and second (2. BoNT) BoNT-A or sham injection. Red rectangles symbolize stepping tests, blue rectangles apomorphine-induced rotation tests.

Here, the experiments were extended by a second intrastriatal BoNT-A injection which followed 6 months after the first one to approach clinical practice using repetitive BoNT-A applications [52,53,54,55,56,57]. Using the method of defining movement IT from videos of AS tests, we studied ITs before and after the induction of hemi-PD in rats, and, furthermore, after a first and second unilateral intrastriatal 1 ng BoNT-A application up to 12 months (Figure 1).

## 2. Results

The results of the IT experiments are divided into two parts: first, the ITs of hemi-PD rats are presented; second, this parameter is tested as to whether it can give new insights into the understanding of the time-limited BoNT-A effects in this animal model.

### 2.1. Initiation Time

#### 2.1.1. Hemi-PD Rats

**Figure 2:**
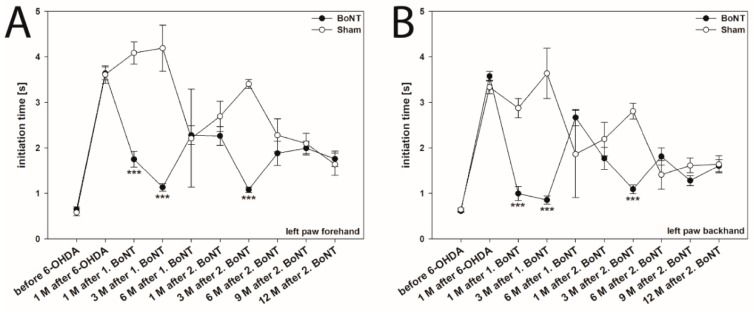

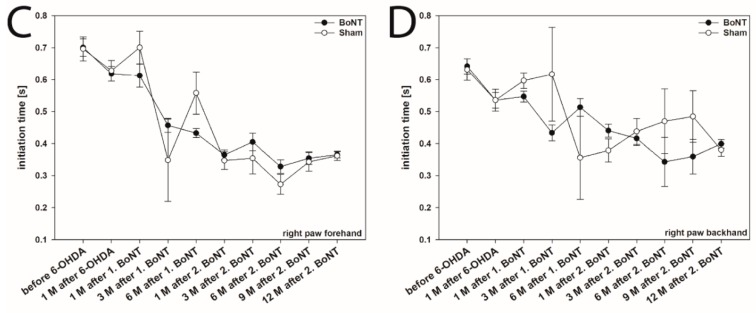
Initiation time of stepping behavior in right side hemiparkinsonian (hemi-PD) rats treated with repetitive intrastriatal BoNT-A or vehicle. Before the 6-OHDA lesion, rats of both experimental groups needed about 0.62 s to start a movement with their left (**A**,**B**) and right (**C**,**D**) forepaws in both forehand and backhand directions. After the 6-OHDA lesion the initiation time in hemi-PD rats was significantly increased in the left forepaw (contralateral to lesion) in both forehand (**A**) and backhand (**B**) directions. Unilateral intrastriatal BoNT-A injection significantly decreased the initiation time of the left forepaw in both forehand (**A**) and backhand (**B**) directions as well as 1 month and 3 months after 1. BoNT-A injection and 1 month after the 2. BoNT-A injection compared to the sham-injected group. The initiation time of movement in the right forepaw in hemi-PD rats in both forehand (**C**) and backhand (**D**) directions were not affected 1 month after lesion nor by the first or second ipsilateral BoNT-A or vehicle injections. Asterisks indicate significant differences compared with the sham group (*** *p* < 0.001). Data are means ± SEM.

In the present study, the IT was evaluated in videos primarily used for the measurement of AS of the forepaws of both body sides in both forehand and backhand directions. Before the 6-OHDA lesion, no significant differences in the ITs for the left and right forelimbs in forehand and backhand directions in either experimental group were observed (left paw forehand: 0.623 ± 0.036 s, left paw backhand: 0.622 ± 0.023 s, right paw forehand: 0.700 ± 0.027 s, right paw backhand: 0.638 ± 0.021 s) (Figure 2A–D).

**Figure 3:**
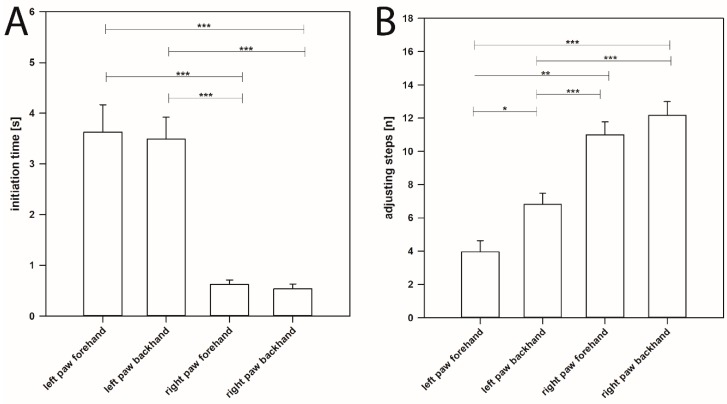
Initiation time of movements (**A**) and adjusting steps (**B**) for the left and right forepaws in stepping test measured 1 month after 6-OHDA injection into the right MFB. (**A**) One month after the 6-OHDA lesion the IT of movements of the left forelimb in both forehand and backhand directions were significantly increased compared to the right forelimb. (**B**) The right side 6-OHDA lesion induced impairments of the adjusting steps of the left forelimb in both forehand and backhand directions. Asterisks indicate significant differences (* *p* < 0.05, ** *p* < 0.01, *** *p* < 0.001). Data are means ± SEM.

One month after 6-OHDA injection, the ITs significantly increased in the left forepaw (contralateral to the 6-OHDA lesion) in forehand (3.629 ± 0.110 s) as well as in backhand (3.493 ± 0.088 s) directions (Figure 2A,B). The respective ITs of left paws in forehand and backhand directions did not differ significantly (forehand: 3.629 ± 0.110 s, backhand 3.493 ± 0.088 s, *p* > 0.05) (Figure 3A).

At the same time, the ITs in the right forepaw, i.e., ipsilateral to the 6-OHDA lesion, were unchanged in both directions (right paw forehand: 0.622 ± 0.018 s, right paw backhand: 0.536 ± 0.019 s) (Figure 2C,D and Figure 3A).

**Figure 4:**
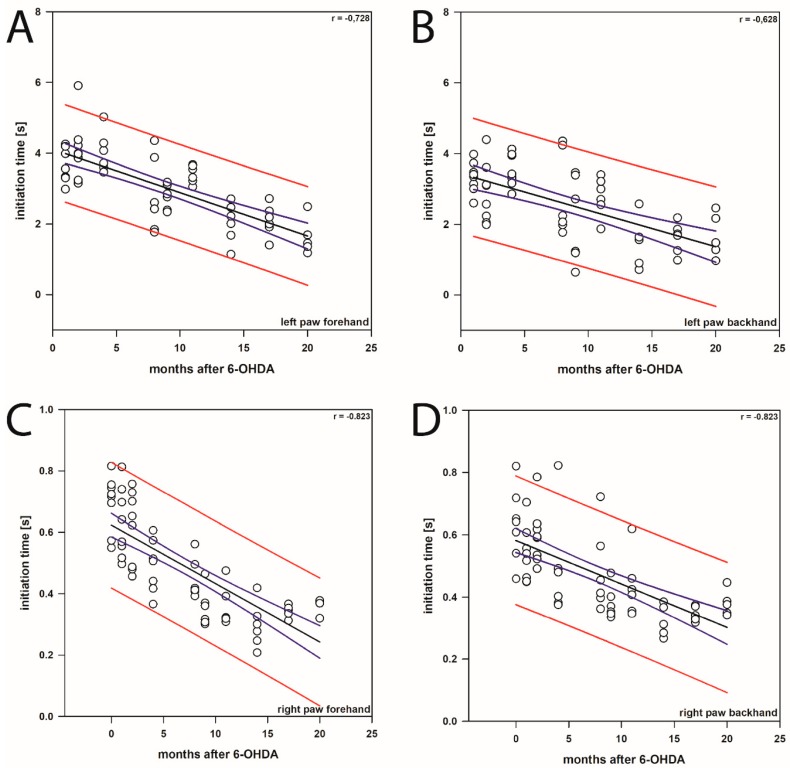
Line/scatter plots of the linear regression of the initiation time of the left (**A**,**B**) and right (**C**,**D**) forepaws in both forehand (**A**,**C**) and backhand (**B**,**D**) directions in sham BoNT-A-injected hemi-PD rats during post-lesion experimental period. The open circles represent the initiation times plotted against the time after the 6-OHDA lesion, the solid black line running through the points represents the regression line, and the colored lines represent the prediction and confidence intervals. The confidence interval for the population (prediction interval, red lines) gives the range of values that define the region containing the population from which the observations were drawn. The confidence interval for the regression line (blue lines) gives the range of values that defines the region containing the true mean relationship between the dependent and independent variables, with the specified level (95%) of confidence.

Interestingly, a linear decrease of the ITs of left forepaws in both forehand and backhand directions was observed in sham BoNT-A-injected hemi-PD rats during the post-lesion experimental period (Figure 2A,B and Figure 4A,B). This decrease in IT was also observed in the right forepaw in forehand and backhand directions (Figure 4C,D). We observed a moderate (Spearman rank correlation coefficient 0.50 to 0.70) or a high negative correlation (correlation coefficient 0.70 to 0.90) of the time after lesion and the ITs (left paw forehand, *n* = 58, *r* = 0.728; left paw backhand, *n* = 58, *r* = 0.628; right paw forehand, *n* = 58, *r* = 0.823; right paw backhand, *n* = 58, *r* = 0.751) (Figure 4A–D).

#### 2.1.2. Hemi-PD Rats Treated with BoNT-A

As compared with the sham-injected group, the first BoNT-A injection into the right CPu reduced, i.e., improved, the IT of the left paw in the forehand direction 1 month (F_1,22_ = 60.539, *p* < 0.001) and 3 months (F_1,21_ = 44.012, *p* < 0.001) after BoNT (Figure 2A). However, this beneficial BoNT-A effect diminished, as 6 months after the first BoNT the IT increased again to values not significantly different from those before BoNT-A (F_1,20_ = 0.0055, *p* = 0.941) (Figure 2A,B). The second BoNT-A application again induced a significant IT reduction of the left paw in the forehand direction 3 months after BoNT-A (F_1,18_ = 416.063, *p* < 0.001) (Figure 2A). As seen after the first BoNT-A injection, the beneficial effect of the second BoNT-A on IT faded with longer survival times, as the increased ITs 6 months (1.884 ± 0.266 s; F_1,18_ = 2.440, *p* = 0.135), 9 months (1.996 ± 0.151 s; F_1,17_ = 0.145, *p* = 0.708), and 12 months (1.763 ± 0.167 s; F_1,14_ = 0.160, *p* = 0.695) after the second BoNT did not differ significantly from the respective sham groups (Figure 2A).

Moreover, reduction of the IT of the left forepaw in the backhand direction was observed 1 month (F_1,22_ = 51.384, *p* < 0.001) and 3 months (F_1,21_ = 34.128, *p* < 0.001) after the first BoNT-A injection, as well as 3 months (F1, 18 = 80.091, *p* < 0.001) after the second intrastriatal BoNT-A (Figure 2B). As seen in the left paw forehand direction, the BoNT-A effect on IT in the left paw backhand direction diminished after longer post injection survival times (Figure 2B).

The comparison of the ITs of the right forepaw both in forehand and backhand directions of the rats before and 1 month after right side 6-OHDA injection revealed no significant differences, nor were the respective ITs affected by the first and second intrastriatal BoNT-A or sham injection for all time points (Figure 2C,D and Figure 3A).

### 2.2. Adjusting Steps

**Figure 5:**
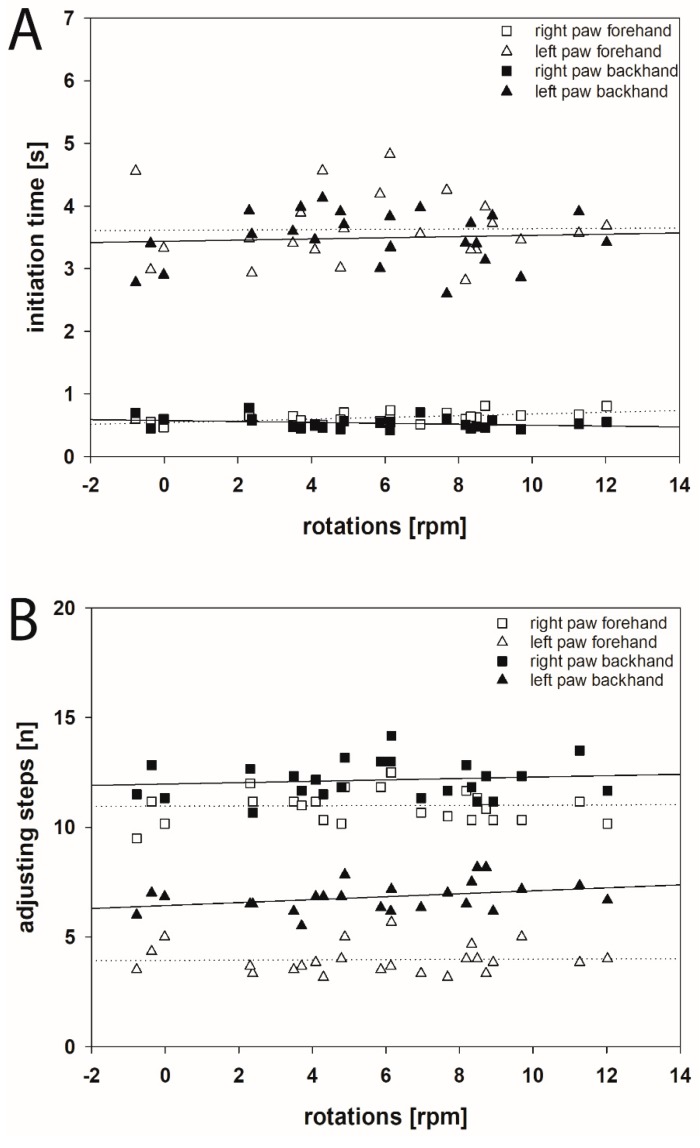
Scatter plots of the initiation time (**A**) and adjusting steps (**B**) in stepping test and apomorphine-induced rotational behavior 1 month after the 6-OHDA lesion. (**A**) No statistically significant correlation between the initiation time of movements of both forelimbs in both forehand and backhand directions and apomorphine-induced rotations after 1 month following the 6-OHDA lesion was found. (**B**) Comparing the adjusting steps for the both forelimbs in forehand and backhand directions with apomorphine-induced rotations 1 month following the 6-OHDA lesion showed no significant correlations. Linear regression lines are displayed for BoNT (solid) and Sham (dotted) groups. For better readability of the figure no error bars are shown.

Before the 6-OHDA lesion, no significant differences in the AS for the left and right forelimbs in forehand and backhand directions in either experimental group were observed (left paw forehand: 9.292 ± 0.201, left paw backhand: 11.701 ± 0.134, right paw forehand: 10.153 ± 0.182, right paw backhand: 11.785 ± 0.113). One month after injection of 6-OHDA into the right MFB, the respective values were: left paw forehand: 3.958 ± 0.136, left paw backhand: 6.813 ± 0.136, right paw forehand: 10.993 ± 0.160, right paw backhand: 12.153 ± 0.175 (Figure 3B and Figure 5B). Neither the first nor the second BoNT-A injection into the CPu significantly changed the number of AS made by either forepaw in either direction [58].

### 2.3. Initiation Time, Adjusting Steps, and Apomorphine-Induced Rotations

#### 2.3.1. Hemi-PD Rats

Comparing the ITs of movements of left and right forepaws both in forehand and backhand directions and apomorphine-induced rotations in individual rats 1 month after induction of hemi-PD revealed that these parameters are not significantly correlated (Figure 5A). Rotational behavior covered a wide range of values (−0.775 to +12.022 rpm; [58]). However, ITs were found rather constant (left paw: 2.600–4.827 s, right paw 0.424–0.813 s). Thus, the IT of forelimb movements is a parameter widely independent from rotational behavior (Figure 5A).

The same holds true for the dependency of AS and apomorphine-induced rotations. Comparing the AS of left and right paws both in the forehand and backhand directions with the number of the respective rotations 1 month after 6-OHDA injection showed that AS and rotations are not significantly correlated (Figure 5B). Values of AS of the left forepaw in hemi-PD rats ranged between 3.167 and 8.167 (left paw forehand: 3.958 ± 0.136, left paw backhand: 6.754 ± 0.163; [58]). Also, the AS of forelimb movements is a parameter independent of the degree of the respective apomorphine-induced rotations (Figure 5B). Moreover, comparing IT and AS data of either forelimb in either direction of identical hemi-PD rats 1 month after 6-OHDA did not reveal a significant correlation (Spearman rank correlation coefficients >0.50).

#### 2.3.2. Hemi-PD Rats Treated with BoNT-A or Sham BoNT-A Injections

**Figure 6:**
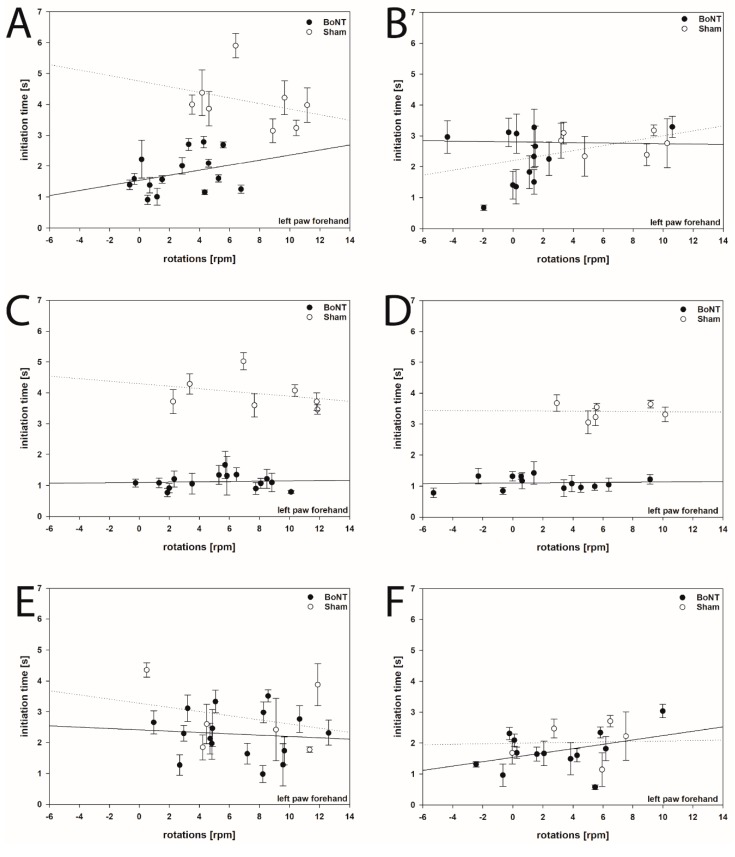
Correlation analysis (Pearson) of the initiation time of stepping movements of the left forepaw (contralateral to the 6-OHDA lesion) in the forehand direction and apomorphine-induced rotations in hemi-PD rats injected intrastriatally with BoNT-A (closed circles) or sham BoNT-A (open circles). No significant correlations between the ITs of the left forepaw in the forehand direction and apomorphine-induced rotations were found 1 month (**A**), 3 months (**C**), and 6 months (**E**) after the first BoNT-A or 1 month (**B**), 3 months (**D**), and 6 months (**F**) after the second BoNT-A. Data represent means ± SEM. Linear regression lines are displayed for BoNT (solid) and Sham (dotted) groups.

Testing for correlations between ITs of left forepaws both in forehand and backhand directions and apomorphine-induced rotations in hemi-PD rats treated with BoNT-A or sham BoNT-A with various post injection survival times revealed no significant correlative dependency of both parameters at any time point (Figure 6A–F). Although BoNT-A both reduced ITs and rotational behavior in hemi-PD rats dependent on post BoNT-A injection survival time [58], seemingly both parameters are differently and independently influenced by intrastriatal BoNT-A.

Further correlation analysis between the IT of stepping movements of both left and right forepaws in forehand and backhand directions measured 1, 3, and 6 months after the first intrastriatal BoNT-A injection and 1, 3, 6, 9, and 12 months after the second intrastriatal BoNT-A injection with the apomorphine-induced rotations are depicted in Figures S1−S8.

## 3. Discussion

Patients in later PD stages have disabling problems with initiation of gate, even when they are still able to accomplish steady-state walking [59,60,61,62,63]. Remarkably, delayed movement initiation, measurable as IT, was found to be the most sensitive parameter in the stepping tests characterizing parkinsonian movement alterations [1,3,4,5,6,25,30,33].

### 3.1. Initiation Time in Hemi-PD Rats According to Olsson et al. (1995)

As the IT seems to be an important and crucial parameter describing the degree of damage in hemi-PD, we focused on a refinement of its determination. The currently and widely used method for testing the IT of stepping for each forelimb was introduced by Olsson et al. [5]. In his experiments, rats had to learn to run spontaneously up a 1.1 m long ramp to their home cage. According to Olsson et al. [5], measurement of the IT starts when the free paw contacts the ramp and ends when the rat initiated the movement with that forelimb. IT testing consists of two tests per day for 3 consecutive days, and the mean of the six tests is calculated. Up to now, the Olsson method has been widely used in hemi-PD rats. Table S1 summarizes studies that measured the IT as a parameter to describe pathological movement behavior in 6-OHDA-induced hemi-PD. Interestingly, in all studies measuring AS (Tables S1 and S2), about one third additionally characterized the IT according to Olsson et al. [5] (Table S1).

### 3.2. Newly Introduced Initiation Time in Hemi-PD Rats

In the present study, we used a new, reliable and simple method, which allows the analysis of the IT of stepping offline from videos taken originally for the measurement of AS. Additionally, we can determine the ITs not only for the rat´s left and right forelimbs comparable with Olsson et al. [5], but additionally in both forelimbs in forehand and backhand directions. The values obtained lay in a very small range, which speaks in favor of an exact determination by using measurements taken from slow motion videos of AS. IT values that have been evaluated in hemi-PD rats using the Olsson method are listed in Table S1. It can be see that they differ considerably not only in naïve rats (1–2 s, [5,31]), but even more in the hampered forelimb of hemi-PD rats (10 s–20 s, [1,30,31,33,34,35,36,37]) up to 60–120 s, [5]).

Before the 6-OHDA lesion, no significant differences in the ITs for either of the forelimbs in the forehand and backhand directions in either experimental group were observed (Figure 2A–D). Principally, this result corroborates the data of Olsson et al. [5] and Pinna et al. [31], although they estimated the IT only “in the direction of the home cage” and not in both forehand and backhand directions. Many others showed IT values after the 6-OHDA lesion merely for the forepaw contralateral to the lesion [33,34,35,64,65] (Table S1). Simultaneously, the IT of the ipsilateral forepaw was unchanged in both forehand and backhand directions, thus confirming others [31,33,34,36,37,64,66].

### 3.3. Comparison to Initiation Time According to Olsson et al.

Compared with the Olsson method, we see several advantages: First, IT is measured from existing videos from experiments assessing the AS. Thus, there is no need for additional experiments with new equipment and separate training and performance of the rats. This reduces the number of experiments and, more importantly, reduces the burden on the animals, enabling a reduction of experiments [67].

Second, the method according to Olsson et al. [5] allows for the measurement of the IT of stepping of left and right forelimbs only in one direction, namely ”in the direction of the home cage”. Our approach evaluates the ITs, comparable with the AS, also in the forehand and backhand directions of both left and right forelimbs. Moreover, the present approach results in exact values for the IT due to the evaluation of slow motion videos from stepping tests.

Distinguishing between ITs in forehand and backhand directions of the affected forelimb seemed interesting, as related AS differed in various studies and in our own experiments; in hemi-PD rats, a higher number of AS was found in the backhand than in the forehand direction [1,30,31,33,38,64,66] (Table S2). Moreover, different therapeutic interventions were described to improve AS either only in the forehand [27] or only in the backhand direction [5,68] of the affected forepaw. Contralateral forehand AS significantly increased after the intraperitoneal administration of L-DOPA in 6-OHDA-lesioned rats [27]. In hemi-PD rats, injection of the DA agonist SKF 38393 restored the number of AS in the backhand direction to a level no longer different from that seen in intact controls [5]. Hemi-PD rats transplanted with DA progenitor cells derived from E13 to E15 displayed a significant improvement of AS in the backhand direction 2 weeks after surgery [68]. In the present study, 1 month after right MFB lesion, the left forelimb showed an AS value of 3.958 ± 0.136 (mean ± SEM) in the forehand direction, but significantly more AS in the backhand direction (6.813 ± 0.136, mean ± SEM) (*p* = 0.024) (Figure 3B and Figure 5B). However, the respective ITs did not differ significantly (*p* = 0.348).

Seemingly, IT as presently defined and IT according to Olsson et al. [5] characterize partly different behavioral aspects of hemi-PD rats. The Olsson rats have to learn and remember to step up the board connected to their home cage, i.e., “running spontaneously up the ramp to the home cage”. If they performed unwanted actions, like returning to the bottom, jumping from the board, or staying on the board, rats were trained until they fulfilled the task [1]. This procedure is attributed to environmental conditional learning, which is mainly controlled by the cerebral cortex and hippocampus [69,70]. These essential learning and memory systems are known to be affected by multitudinous factors, not only by the dopaminergic system [30].

However, the motor initiation deficits of the forelimbs are primarily influenced by DA depletion [71,72,73]. As the recommended IT does not require the rat to perform a previously learned task, we assume that the present IT is a result of a spontaneous voluntary initiation of movements in the basal ganglia loops.

### 3.4. Initiation Time in Hemi-PD Rats During Post-Lesion Survival

Up until now, the ITs of forelimb movements in hemi-PD rats were evaluated up to 6 months post lesion [74]. Most studies, however, analyzed the IT only a few days or 3–5 weeks after the 6-OHDA lesion ([1,30,31,33,34,35,36,37,64,65,66]; for details see Table S1).

Here, we measured ITs for about 20 months after induction of hemi-PD. Interestingly, ITs of both forelimbs in both forehand and backhand directions significantly decreased from 1 month post-lesion onwards, i.e., the rats obviously behaviorally improved motor performance over time (Figure 4A–D). Seemingly, rats that were re-tested eight times during the 20 months period learned more quickly to initiate movement of the respective paws. Recovery of brain function is a well-documented phenomenon [75,76,77,78] and is known to be influenced by various factors, among which, besides training, are size of the lesion, testing post-lesion delay and age at which the lesion was sustained [78,79,80,81]. The beneficial effects of postoperative, repeated training on recovery of function has also been reported following spinal cord transection in cats [82], lesion of the hippocampus [83], ischemic infarct [84], and neuronal transplants [85]. Previous studies by Abrous et al. [86] reported that 6-OHDA-lesioned rats may learn “how to perform test during the manipulation and pretesting procedure”. The trend to improvement in IT tests over time was already seen, but not specifically mentioned in the paper of Olsson et al. [5]: ITs in both their control and in the hemi-PD rats decreased during the test period, although the animals were tested only during 3 weeks after the 6-OHDA lesion [5]. Interestingly, this learning effect caused by repetitive training was even obvious in the right paw. The IT values of this paw 4 weeks after right side MFB lesion did not differ from the prelesion data, but also decreased later on (Figure 4C,D).

### 3.5. Initiation Time in Hemi-PD Rats Following Intrastriatal BoNT-A Injection

We used this novel method to study the possibly beneficial effect of repeated unilateral intrastriatal BoNT-A application on delayed movement initiation in hemi-PD rats. Injection of 1 ng BoNT-A into the right striatum, i.e., ipsilateral to the 6-OHDA lesion, significantly improved the IT of the contralateral forepaw in hemi-PD rats 1 month and 3 months after BoNT-A, but 6 months after BoNT-A the IT returned to the primal pathological level (Figure 2A,B). Three months after a second intrastriatal BoNT-A injection, IT decreased again, followed by a recurrent impairment that lasted up to a further 12 months (Figure 2A,B). Concerning the time-dependent outcome of BoNT-A application, these findings correspond to our prior results in an apomorphine-induced rotation test: injection of BoNT-A into the CPu of hemi-PD rats reduced apomorphine-induced rotation behavior significantly for at least 3 months [38,39,40,42,43,44,87]. In addition, compared with sham injections, a second BoNT-A again significantly decreased apomorphine-induced rotations, whereas the beneficial effect faded with longer post-injection survival times [58].

### 3.6. Correlating Initiation Time, Adjusting Steps, and Apomorphine-Induced Rotations

There is still controversy whether spontaneous motor behavior like AS and IT and drug-induced rotational behavior are related, or the various parameters define different behavioral aspects of the rat resulting from the unilateral lack of dopamine [33,88]. Most publications only show a relationship between the behavioral impairments in stepping tests with damage to the mesostriatal dopaminergic pathway. There was a relationship between the AS with the level of DA neuron depletion [3,89,90] and striatal TH-ir fiber density [91,92,93] Thus, Kirik et al. [3] showed that a 60%–70% reduction in TH-ir fiber density in the lateral striatum, accompanied by a 50%–60% reduction in TH-ir cells in SNpc, was sufficient for the induction of a significant impairment in initiation of stepping [3]. Two of the behavioral measures, i.e., contralateral forelimb stepping and paw use in the paw reaching test, showed good correlations with both the nigral TH-ir cell number and striatal TH-ir fiber density (r values between 0.51 and 0.74). Respective scatter diagrams suggest that the relationship between forelimb stepping scores or successful reaches in the staircase test, and TH-ir cell number or TH-ir fiber density may conform to an S-shaped curve. A reduction of TH-ir cell bodies in SNpc or TH-ir fiber density in the whole striatum by at least 50% was necessary for the induction of any measurable deficit in forelimb stepping. The reduction in forelimb stepping scores was well correlated with the extent of cell loss and degree of striatal TH-ir fiber innervation. A 50% reduction in forelimb stepping scores was seen in animals with approximately 60% loss of nigral cells and total striatal TH-ir fiber density.

Different levels of mesencephalic DA denervation 2 weeks after injecting 6-OHDA in dosages of 4, 6, and 8 µg into the MFB affected forelimb akinesia examined by assessing stepping [89]. Only animals that had a DA lesion > 65% in the SNpc displayed marked changes in the number of AS. A significant deficit in AS performance could be observed only following ~75% and ~55% reduction of SNpc and ventral tegmental area (VTA) DA neurons, respectively. Although 4 µg of 6-OHDA reduced the number of TH-ir neurons in the SNpc by 60%, both stepping test performance and neuronal activity in the substantia nigra pars reticularis remained indistinguishable from control animals. By contrast, animals that received 6 µg of 6-OHDA showed a marked reduction of TH-ir cells in the SNpc (75%) and VTA (55%), and a significant stepping deficit. The changes were not further enhanced with 8 µg of 6-OHDA, a dose that induced an extensive DA lesion (>95%) in the SNpc and 70% reduction of DA neurons in the VTA [89]. Barneoud and colleagues (2000) examined the behavioral consequences of a partial unilateral dopaminergic denervation by striatal injection of 6-OHDA [90]. Animals were impaired exclusively for the paw contralateral to the side of the lesion in staircase tests, but were able to performed normal AS with the impaired paw, indicating that the partial lesion induced a coordination deficit of the paw rather than a deficit of movement initiation. After a complete lesion, stepping adjustments of the contralateral paw were dramatically impaired (by about 80%). A variability in AS outcomes was found, reflecting the extent of cell loss in the ipsilateral SNpc [92,93]. Loss of TH-ir cells in SN was ~60% in the moderately impaired animals, and >80% in the severely impaired ones. The overall correlation between the rats´ AS performance test and the TH-ir cell number in the SN was highly significant [92,93].

Few publications have investigated the dependency of the IT of forelimb movements and the number of adjusting footsteps after unilateral striatal 6-OHDA lesions in rats [1,3,30,66]. One or two striatal injections of 6-OHDA (single or two-site terminal lesions) had only small effects on the initiation of forelimb stepping: 6.5–8.8 steps of the contralateral paw, compared with 10–12 steps of the intact ipsilateral paw [3]. More pronounced deficits were seen in animals that had received three or four intrastriatal injections or injections at the junction between globus pallidus and CPu. In these groups, initiation of stepping was reduced by about 60–65% at 3 weeks post lesion, and in all groups the impairments remained stable at 8 weeks post lesion [3]. Sun et al. [66,74] and Fang et al. [1,30] compared IT of a single-site intrastriatal 6-OHDA lesion (1-lesion), four-site lesion (4-lesion) and MFB injection. In all models, the IT of stepping was significantly longer in the affected forelimb compared with the normal side; however, measurements did not differ between groups. At the same time, AS both in the forward and backward directions was significantly reduced in the affected forelimb compared with the normal side, but again no differences were observed among the 1-lesion, 4-lesion, and MFB groups [1,30,66,74].

Concerning the hemi-PD mouse model after injection of 6-OHDA into the SNpc, Grealish et al. [94] correlated the outcome of drug-induced rotation tests with concurrent AS, forepaw use and lateral sensorimotor integration. The impairments seen in AS showed no correlation with striatal denervation and only very weak correlation with the TH-ir cell loss. Mice with a decreasing number of AS presented an increasing apomorphine-induced rotational behavior. The corridor task and apomorphine-induced rotation showed the best correlation: increasing neglect in the corridor task correlated with increasing apomorphine-induced rotation [94].

In hemi-PD rats, Badstuebner et al. [33] found the pathologically increased IT (method of Olsson et al. 1995) [5] of the contralateral forepaw reduced by deep brain stimulation of the subthalamic nucleus. At the same time, rotational behavior was not consistently reduced by this unipolar stimulation.

Limitations of the impact of the apomorphine-induced rotations test were also discussed by Metz and Whishaw [88]. They correlated the apomorphine-induced rotations with the spontaneous motor tests, skilled forelimb reaching task and skilled horizontal ladder rung walking task and found that different measurements of behavior did not reflect the results obtained in drug-induced rotation.

In the present study, the AS of left and right paws both in the forehand and backhand direction and apomorphine-induced rotations in individual rats 1 month after induction of hemi-PD are not significantly correlated. Nevertheless, in hemi-PD rats AS of movements of the left forepaws in the forehand direction differed significantly from those of the backhand direction, hemi-PD rats showing more AS in backhand direction. These results corroborate findings that can be deduced from the figures of others [1,3,31,33,34,64,66,90,95,96,97]. Also Köllensperger et al. [98] and Winkler et al. [91] only mentioned this phenomenon without any explanation. Lettfuss et al. [99] explained the significant difference in the direction of AS by higher impact of reflex circuits when the extremity is moved in a backhand direction. The same phenomenon was discussed as caused by minor proprioceptive stimulation in the forehand direction compared to backhand, which causes fewer reflex movements [100].

Correlating our present results of AS and IT of movements from stepping tests with the apomorphine-induced rotation behavior of the identical hemi-PD rats both sham-treated or BoNT-A-treated [58] revealed that there were no significant correlations between these three parameters. Neither AS nor IT were significantly correlated with apomorphine-induced rotations, nor were AS and IT significantly related (Spearman rank correlation coefficients >0.50). Corroborating others, it can be concluded that AS, IT, and apomorphine-induced rotations represent widely independent parameters describing the behavior of hemi-PD rats, and that after therapeutic interventions as many parameters as possible should be investigated to judge the induced effects.

## 4. Materials and Methods

### 4.1. Animals

Young, adult, 3 month old, male Wistar rats (strain Crl:WI BR) obtained from Charles River Wiga (Sulzfeld, Germany) weighing 295–305 g at the time of the first surgery were used for this study. Animals were housed in standard cages in a temperature-controlled room (22 °C ± 2 °C) under a 12 h light/dark cycle, with access to food and water ad libitum. All procedures used in the present study complied with the guidelines on animal care. All experiments were approved by the State Animal Research Committee of Mecklenburg-Western Pomerania (LALLF M-V 7221.3-1.1-003/13 from 26 April 2013). For determining the IT described in this paper, videos were systematically reevaluated from stepping experiments from animal series previously published [58].

### 4.2. Induction of Hemiparkinsonism

Rats were deeply anesthetized via intraperitoneal injection of a mixture of ketamine (50 mg/kg BW) and xylazine (4 mg/kg BW). All surgeries were carried out under aseptic conditions. In order to cause a lesion of the dopaminergic nigrostriatal pathway, a unilateral injection of 24 µg 6-OHDA (Sigma-Aldrich, St. Louis, MO, USA) dissolved in 4 µL of 0.1 M citrate buffer was performed over 4 min via a 26 gauge 5 µL Hamilton syringe into the right MFB using a David Kopf stereotactic frame. The injection coordinates with reference to bregma were: AP = −2.3, L = 1.5 to the right, V = −9.0 [101].

### 4.3. Injection of BoNT-A into the Striatum

Six weeks after the 6-OHDA lesion, animals underwent a second stereotactic surgery. Hemi-PD rats were treated with either BoNT-A (*n* = 16) or vehicle (*n* = 8). 2 × 1 µL BoNT-A solution (Lot No. 13028A1A; List, Campbell, CA, USA; purchased via Quadratech, Surrey, UK; BoNT-A dissolved in phosphate-buffered saline (PBS) supplemented with 0.1% bovine serum albumin (BSA)) was injected with a total dose of 1 ng BoNT-A into the right CPu at two sites [38,39,40,87]. The respective coordinates with reference to bregma were: AP = +1.3/−0.4 mm, L = 2.6/3.6 mm to the right, and V = −5.5 mm [101]. At each injection site 1 µL (= 0.5 ng) of BoNT-A solution was delivered over 4 min using a 26 gauge 5 µL Hamilton syringe. Sham BoNT-A rats received 2 × 1 µL PBS + 0.1% BSA. Animals underwent a second BoNT-A or sham injection with vehicle solution 6 months after the first one, respectively [58].

### 4.4. Behavioral Testing

An apomorphine-induced rotation test and a stepping test were performed before the 6-OHDA lesion, 4 weeks thereafter, and 1, 3, and 6 months after the first injection of BoNT-A or sham BoNT-A into the dopamine-depleted striatum, as well as 1, 3, 6, 9, and 12 months after the second BoNT-A or vehicle intrastriatal injection.

#### 4.4.1. Apomorphine-Induced Rotation Test

Four weeks after 6-OHDA injection, the success of the lesion was verified by the apomorphine-induced rotation test according to Ungerstedt and Arbuthnott [9]. All 6-OHDA-injected rats (*n* = 24), irrespective of the displayed apomorphine-induced rotation rate, were included in this study. Starting 5 min after the injection of apomorphine (0.25 mg/kg, i.p.; Teclapharm, Germany), the animals’ turns were registered in a self-constructed automated rotometer device over 40 min. Rotations were analyzed as complete 360° turns, and mean rotations per minute (rpm) were calculated (anti-clockwise: +, clockwise: −).

#### 4.4.2. Stepping Test

• Adjusting Steps

The stepping test, evaluating akineasia/bradykinesia, was performed as originally described by Olsson et al. in 1995 [5]. Rats were handled by the experimenter during 3 days to allow animals to familiarize themselves with the experimenter’s grip and with the test procedure [5,33,102]. Thereafter, AS were registered twice per day on three consecutive days. Briefly, the rat was held by the investigator with one hand gently blocking both its hind limbs and the non-monitored forelimb, the unrestrained forepaw touching the table. Then the rat was moved slowly sideways across the table (90 cm in 5 s) and the number of AS of the respective unrestrained left or right forepaw was counted while moving both in the forehand and backhand directions. The sequence of testing was: left forelimb forehand, left forelimb backhand, right forelimb forehand, right forelimb backhand. All the sessions were video-recorded to allow counting of the number of AS afterwards by an investigator blinded to the state of the rats. Finally, the respective means were calculated.

• Initiation Time

We measured the IT of movements using the same video-recorded sessions as for the evaluation of the AS. Here, we define the IT as the time period between (i) when the experimenter starts to move the rat in forehand or backhand directions, and (ii) the moment the rat starts side stepping with the respective freely movable forelimb. Mean values were calculated from the 6 experiments for 4 different initiation times, i.e., (i): left forelimb forehand, (ii) left forelimb backhand, (iii) right forelimb forehand, (iv) right forelimb backhand. We employed the VLC media player program and its extension Jump to time, previous frame v 2.1.” [103], which made it possible to determine very precisely the time point of the stepping initiation.

### 4.5. Data Analysis

The results were presented as means ± SEM. In all cases, *p* values ≤ 0.05 were considered significant and *p* values <0.01 were considered highly significant. Data from the stepping test were subjected to two-way repeated measures ANOVA using SigmaPlot 14 software (Systat Software, Inc., San Jose, CA 95110, USA). To measure the strength of the association between initiation time, adjusting steps and apomorphine-induced rotation data, Pearson Product Moment Correlation tests were done using SigmaPlot 14 software. If residuals (distances of the data points from the regression line) are not normally distributed with a constant variance, Spearman Rank Order Correlation tests were done.

## 5. Conclusions

First, in the present study we describe a new method which enables the evaluation of the initiation time of movements for both forelimbs both in forehand and backhand directions. This establishes a possibility to evaluate exactly the initiation time of movements as an index for motor initiation deficits and akinesia, including therapeutic interventions. Second, the data acquisition requires no further experiments and, therefore, a reduction of the number of experiments is realized. Third, in hemi-PD rats unilateral BoNT-A injection into the CPu improved the deficits of the initiation time of the contralateral forelimbs for a limited time window of 3 months after both the first BoNT-A and the second BoNT-A application. Fourth, adjusting steps, initiation time and apomorphine-induced rotations, all studied in the same animals, proved to be independent parameters characterizing different aspects of behavior of hemi-PD rats.

## Supplementary Materials

The following are available online at https://www.mdpi.com/1422-0067/20/4/992/s1, Figures S1–S8; Tables S1–S2.

## Abbreviations

6-OHDA	6-hydroxydopamine
AS	adjusting steps
BoNT-A	botulinum neurotoxin-A
BW	body weight
CPu	caudate-putamen (striatum)
DA	dopamine
hemi-PD	hemiparkinsonian
ir	immunoreactive
IT	initiation time
M	month
MFB	medial forebrain bundle
PD	Parkinson’s disease
rpm	rotations per minute
SNpc	substantia nigra pars compacta
TH	tyrosine hydroxylase
VTA	ventral tegmental area
