# Supplementary material for: Botulinum Neurotoxin-A Injected Intrastriatally into Hemiparkinsonian Rats Improves the Initiation Time for Left and Right Forelimbs in Both Forehand and Backhand Directions
Source: Int J Mol Sci. 2019 Feb 25;20(4):992. doi: 10.3390/ijms20040992 (PMC6412467; doi:10.3390/ijms20040992)
Supplement: Supplementary file 1 [file ijms-20-00992-s001.pdf]

International Journal of *Molecular Sciences*

MDPI

Supplement

Botulinum Neurotoxin-A Injected Intrastriatally into Hemiparkinsonian Rats Improves the Initiation Time for Left and Right Forelimbs in Both Forehand and Backhand Directions

Veronica Antipova [1,2], Carsten Holzmann [3,4], Alexander Hawlitschka [1] and Andreas Wree [1,4,*]

1 Institute of Anatomy, Rostock University Medical Center, Rostock, D-18057 Rostock, Germany; veronica.antipova@medunigraz.at (V.A.); alexander.hawlitschka@med.uni-rostock.de (A.H.)
2 Gottfried Schatz Research Center for Cell Signaling, Metabolism and Aging, Macroscopic and Clinical Anatomy, Medical University of Graz, A-8010 Graz, Austria
3 Institute of Medical Genetics, Rostock University Medical Center, Rostock, D-18057 Rostock, Germany; carsten.holzmann@med.uni-rostock.de
4 Centre of Transdisciplinary Neuroscience Rostock, Rostock, D-18147 Rostock, Germany
* Correspondence: andreas.wree@med.uni-rostock.de; Tel.: +49-381-494-8429

1. Supplement

Table 1. List of publications of stepping test evaluating adjusting steps of the forepaws and measuring the "classical" initiation time according to Olsson et al. (1995) in hemi-parkinsonian rats after 6-OHDA lesion. Abbreviations: b – backhand, contra – contralateral, f – forehand, ipsi – ipsilateral, not done – experiment not done, not shown – experiment done, but result not conveyed, S-D - Sprague-Dawley rat, W – Wistar rat.

Publication	Strain, gender	Site of 6-OHDA<br>Dosage of 6-OHDA<br>Pretreatment with desipramine	Adjusting steps, initiation time, time after lesion	Adjusting steps (number of steps) mean ± SEM or SD	Initiation time in seconds (s) mean ± SEM or SD
Antala et al. 2012 [1]	W, m	- Right striatum or right SN<br>- 7 µg 6-OHDA<br>- not done	3 weeks	6-OHDA:<br>contra (f) 4.50 ± 0.56, (b) 5.33 ± 0.87; ipsi not shown<br>Sham:<br>contra (f) 10.67 ± 0.88, (b) 11.83 ± 1.19; ipsi not shown	6-OHDA:<br>contra 17.17 ± 1.8; ipsi not shown<br>Sham:<br>contra 4 ± 0.58; ipsi not shown

Badstuebner et al. 2017 [2]	W, m	- Right MFB<br>- 24 µg 6-OHDA<br>- not done	12-14 days	6-OHDA:<br>contra stepping bias (%) (f) approx. 10%, (b) approx. 29%; ipsi not shown<br>Sham:<br>contra stepping bias % (f) approx. 50%, (b) approx. 51%; ipsi not shown	6-OHDA:<br>contra approx. 8; ipsi approx. 3<br>Sham:<br>contra approx. 1,5; ipsi approx. 1
Fang et al. 2006a [3]	S-D, m	- Lateral sector of the striatum (one or four lesions)<br>- 7 µg 6-OHDA<br>- not done	3 weeks	6-OHDA:<br>contra (f) approx. 1.7, (b) approx. 8; ipsi not shown<br>Sham: not done	6-OHDA:<br>contra approx. 25 (one lesion), approx. 30 (four lesion); ipsi not shown<br>Sham: not done
Fang et al. 2006b [4]	S-D, m	- Right striatum (one to four lesions)<br>- 7 µg 6-OHDA<br>- not done	3 weeks	6-OHDA:<br>contra (f) approx. 1.8 (one lesion), (f) approx. 1.9 (four lesion), (b) approx. 5.8 (one lesion), (b) approx. 5.9 (four lesion); ipsi (f) approx. 7 (one lesion), (f) approx. 7.2 (four lesion), (b) approx. 11,8 (one lesion), (b) approx. 11 (four lesion)<br>Sham: not shown	6-OHDA:<br>contra approx. 19 (one lesion-four lesion); ipsi approx. 3<br>Sham: not shown
Fang et al. 2010 [5]	S-D, m	- Lateral sector of the striatum (one or four lesions)<br>- 7 µg 6-OHDA<br>- not done	3 weeks	6-OHDA:<br>contra (f) approx. 1 (one lesion), (f) approx. 1 (four lesion), (b) approx. 8 (one lesion), (b) approx. 7.5 (four lesion); ipsi not shown<br>Sham: not done	6-OHDA:<br>contra approx. 28 (one lesion), approx. 34 (four lesion); ipsi not shown<br>Sham: not done
Mukhida et al. 2001 [6]	W, f	- Right ascending mesostriatal dopaminergic pathway<br>- 3.6 µg 6-OHDA<br>- not done	3 weeks	6-OHDA:<br>contra (f) approx. 3; ipsi (f) approx. 28<br>Sham: not done	6-OHDA:<br>contra approx. 20; ipsi approx. 2<br>Sham: not done

Olsson et al. 1995 [7]	S-D, f	- Right ascending mesostriatal dopamine pathway<br>- 3.6 µg 6-OHDA<br>- not done	3-5 weeks	6-OHDA:<br>contra (f) approx.2, (b) approx. 1-2; ipsi (f) approx. 12, (b) approx. 12<br>Sham: not done	6-OHDA:<br>contra approx. 60-120; ipsi approx.1-2<br>Sham: not done
Pinna et al. 2007 [8]	S-D, m	- Left MFB<br>- 8 µg 6-OHDA<br>- 10 mg/kg	2-3 weeks	6-OHDA:<br>contra (f) approx. 2 (2W) and 1 (3W) weeks after lesion, (b) approx. 3 (2W) and 4 (3W) after lesion; ipsi (f) approx. 7 (2W) and 6 (3W) weeks after lesion (b) approx. 9 (2W) and 8 (3W) weeks after lesion<br>Sham: not done	6-OHDA:<br>contra approx. 5 (2 W) and 10 (3W) weeks after lesion; ipsi approx. 1 (2 W and 3W) weeks after lesion<br>Sham: not done
Pinna et al. 2010 [9]	S-D, m	- Left MFB<br>- 8 µg 6-OHDA<br>- 10 mg/kg	4 weeks	6-OHDA:<br>contra (f) approx. 2 (2W) and 1 (3W) weeks after lesion, (b) approx. 3 (2W) and 4 (3W) weeks after lesion; ipsi not shown<br>Sham: not done	6-OHDA:<br>contra approx. 7 (2W) 15 (3W) weeks after lesion; ipsi not shown<br>Sham: not done
Singh et al. 2006a [10]	S-D, f	- Lateral striatum<br>- 8 µg 6-OHDA<br>- not done	5 weeks	6-OHDA:<br>contra (f) 6 ± 1, (b) 7 ± 2; ipsi not shown<br>Sham: not done	6-OHDA:<br>contra 16 ± 2; ipsi not shown<br>Sham: not done
Singh et al. 2006b [11]	S-D, f+m	- Striatum<br>- 8 µg 6-OHDA<br>- not done	4-5 weeks	6-OHDA:<br>contra (f) 4 ± 1, (b) 5 ± 2; ipsi not shown<br>Sham:<br>contra (f) 12 ± 1, (b) 13 ± 2; ipsi not shown	6-OHDA:<br>contra 15 ± 1; ipsi not shown<br>Sham:<br>contra 2 ± 1; ipsi not shown
Sun et al. 2010 [12]	S-D, m	- Striatum (one or four lesions) or MFB<br>- 7 µg 6-OHDA<br>- not done	3 weeks	6-OHDA:<br>contra (for one, four, MFB lesions) (f) approx. 2, (b) approx. 4; ipsi (for one, four, MFB lesions) (f) approx. 6-7, (b) approx. 11-12<br>Sham: not shown	6-OHDA:<br>contra (for one, four, MFB lesions) approx. 20; ipsi (for one, four, MFB lesions) approx. 5<br>Sham: not shown

Sun et al. 2013 [13]	S-D, m	- Striatum (one or four lesion) or MFB<br>- 7 µg 6-OHDA<br>- not done	1 and 6 months	6-OHDA:<br>contra (for one, four, MFB lesions) (f) approx. 2, (b) approx. 4; ipsi (for one, four, MFB lesions) (f) approx. 6,<br>(b) approx. 12<br>Sham: not shown	6-OHDA:<br>contra (for one, four, MFB lesions) approx. 20; ipsi approx. 5<br>Sham: not shown
Zhang et al. 2016 [14]	S-D, m	- MFB<br>- 6 µl 6-OHDA<br>- not done	4 weeks	not done	6-OHDA:<br>contra approx. 80; ipsi not shown<br>Sham:<br>contra approx. 1; ipsi not shown
Present publication (newly defined initiation time)	W, m	- right MFB<br>- 24 µg 6-OHDA<br>- not done	4 weeks	6-OHDA:<br>contra (f) 3.958 ± 0.136, (b) 6.813 ± 0.136; ipsi (f) 10.993 ± 0.160, (b) 12.153 ± 0.175<br>Sham: not done	6-OHDA:<br>contra (f) 3.629 ± 0.110, (b) 3.493 ± 0.087; ipsi (f) 0.622 ± 0.018, (b) 0.536 ± 0.019<br>Sham: not done

Table 2. List of publications evaluating adjusting steps of the forepaws in hemi-parkinsonian rats after 6-OHDA lesion. Abbreviations: b – backhand, contra – contralateral, f – forehand, ipsi – ipsilateral, not done – experiment not done, not shown – experiment done, but result not conveyed, S-D - Sprague-Dawley rat, W – Wistar rat.

Publication	Strain, gender	Site of 6-OHDA<br>Dosage of 6-OHDA<br>Pretreatment with desipramine	Adjusting steps, time after lesion	Adjusting steps, number of steps of contralateral and ipsilateral paw<br>mean ± SEM or SD
Abedi et al. 2013 [15]	S-D, m	- Right GP<br>- 12.5 μg 6-OHDA<br>- 25 mg/kg	4 weeks	6-OHDA:<br>contra (f) 1.42 ± 0.32; ipsi (f) 5.25 ± 0.22<br>Sham:<br>contra (f) 5.25 ± 0.32; ipsi (f) 5.23 ± 0.34
Acuña-Lizama et al. 2013 [16]	W, m	- Right SNpc<br>- 10.5 μg 6-OHDA<br>- 25 mg/kg	11-12 weeks	6-OHDA:<br>contra (f) 0.5 ± 0.2 (Gr. A, caffeine after lesion) and 13.5 ± 0.3 (Gr. B, theophylline after lesion); ipsi (f) 13.4 ± 0.3 (Gr. A) and 1.9 ± 1.0 (Gr. B)<br>Sham: not done
Antipova et al. 2017 [17]	W, m	- Right MFB<br>- 24 μg 6-OHDA<br>- not done	4 weeks	6-OHDA:<br>contra (f) 3.95 ± 0.13, (b) 6.70 ± 0.17; ipsi (f) 10.93 ± 0.13, (b) 12.02 ± 0.13<br>Sham:<br>contra (f) 3.98 ± 0.19, (b) 7.04 ± 0.24; ipsi (f) 11.13 ± 0.18, (b) 12.42 ± 0.18
Baker et al. 2000 [18]	W, f	- Right ascending nigrostriatal pathway<br>- 3.6 μg of 6-OHDA<br>- not done	2 weeks	6-OHDA:<br>contra approx. 4; ipsi approx. 24 (total number in both forehand and backhand directions)<br>Sham: not done
Barneoud et al. 2000 [19]	OFA, f	- Experiment 1 and 2 – striatum<br>Experiment 3 – MFB<br>- 10 und 20 μg 6-OHDA<br>- not done	1 and 6 weeks	6-OHDA (complete lesion, MFB):<br>contra (f) approx. 4, (b) approx. 11; ipsi (f) approx. 13, (b) approx. 13<br>Sham:<br>contra (f) approx. 13, (b) approx. 13, ipsi (f) approx. 13, (b) approx. 13

Betts et al. 2011 [20]	S-D, m	- above the SNpc<br>- 12 µg 6-OHDA<br>- 25 mg/kg desipramine and 5 mg/kg pargyline	6 days	6-OHDA: use of contra paw (% of pre-lesion)<br>contra (f) approx. 50%, (b) approx. 40%; ipsi not done<br>Sham: not done
Bordia et al. 2015 [21]	S-D, m	- MFB<br>- 3 µg 6-OHDA<br>- not done	2 to 8 weeks	6-OHDA: number of total steps (100 %)<br>contra (f) approx. 23%; ipsi not done<br>Sham: not done
Cerri et al. 2015 [22]	W, m	- Right striatum<br>- 20 µg 6-OHDA<br>- not done	1 day	6-OHDA:<br>contra (f) approx. 2-3; ipsi not done<br>Sham:<br>contra (f) approx. 10-12; ipsi not done
Chotibut et al. 2017 [23]	S-D, m	- Right MFB<br>- 16 µg 6-OHDA<br>- not done	1, 7 and 17 days	6-OHDA: as group mean lesioned forepaw stepping (%)<br>contra (f) approx. 30 %; ipsi not done<br>Sham:<br>ipsi (f) approx. 100%; contra not done
Dowd et al. 2005 [24]	Lister Hooded, f	- MFB<br>- 12 µg 6-OHDA<br>- not done	2 weeks	6-OHDA :<br>contra (f) approx. 2; ipsi not shown<br>Sham: not done
Dowd and Dunnett 2005 [25]	Lister Hooded, m	- MFB or striatum<br>- MFB<br>- 12 µg 6-OHDA<br>- Striatum<br>- 28 µg 6-OHDA<br>- not done	4 months	6-OHDA:<br>MFB contra (f) approx. 2.5, ipsi not shown<br>Striatum contra (f) approx. 3; ipsi not shown<br>Sham: not done
Frau et al. 2017 [26]	S-D, f + m	- Right MFB<br>- 16 µg 6-OHDA<br>- not done	3 and 4 weeks	6-OHDA:<br>contra (f) approx. 1-5; ipsi (f) approx. 20<br>Sham: not done
Hahn et al. 2009 [27]	S-D, f	- Right mesostriatal pathway<br>- 10.8 µg 6-OHDA<br>- not done	14 days and 6 weeks	6-OHDA:<br>contra (b) approx. 2-4, ipsi not done<br>Sham: not done

Ivanova et al. 2015 [28]	W, m	- Left MFB<br>- 12 µg 6-OHDA<br>- not done	7, 14, 21 days	Ratio of the numbers of steps made by the impaired forelimb to the total number of steps made by both forelimbs (%).<br>6-OHDA:<br>contra (f) 20.00 ± 7.45; ipsi not done<br>Sham:<br>contra (f) 46.90 ± 3.50; ipsi not done
Kim et al. 2011 [29]	S-D, m	- Right MFB<br>- 25 µg 6-OHDA<br>- not done	4 weeks	6-OHDA:<br>contra (f) approx. 2; ipsi (f) approx. 13<br>Sham:<br>contra (f) approx. 13; ipsi (f) approx. 13
Kirik et al. 1998 [30]	S-D, f	- Right striatum or MFB<br>- Right striatum<br>- 20 µg 6-OHDA<br>- MFB<br>- 13.5 µg 6-OHDA<br>- not done	3 and 8 weeks	6-OHDA:<br>Striatum: contra (f) 6.5 ± 1.1; ipsi (f) 10.6 ± 1.1<br>MFB: contra (f) 1.2 ± 0.4; ipsi (f) 9.5 ± 1.6<br>Sham<br>contra (f) 10.9 ± 0.6; ipsi (f) 10.6 ± 0.3
Kirik et al. 2000 [31]	S-D, f	- Right striatum<br>- 20 µg 6-OHDA<br>- not done	4 and 6 weeks	6-OHDA:<br>contra (f) approx. 3-7; ipsi (f) approx. 11-13<br>Sham: not done
Kirik et al. 2001a [32]	S-D, f	- Right striatum<br>- 28 µg 6-OHDA<br>- not done	4 weeks	6-OHDA:<br>contra (f) approx. 1- 4; ipsi not shown<br>Sham: not done
Kirik et al. 2001b [33]	S-D, f	- Right striatum<br>- 28 µg 6-OHDA<br>- not done	1, 5, 7 and 11 weeks	6-OHDA:<br>contra (f) approx. 2; ipsi not shown<br>Sham: not done
Köllensperger et al. 2007 [34]	W, m	- Left MFB<br>- 8 µg 6-OHDA<br>- not done	4 weeks	6-OHDA:<br>contra (f) 0.72 ± 1.24, (b) 10.42 ± 1.38; ipsi (f) 6.63 ± 4.28, (b) 12.88 ± 1.68<br>Sham:<br>contra (f) 9.8 ± 3.80, (b) 13.67 ± 1.07; ipsi (f) 11.2 ± 11.74, (b) 13.17 ± 0.89

Lettfuss et al. 2012 [35]	S-D, m	- Left MFB<br>- 12 μg 6-OHDA<br>- 20 mg/kg	22 - 24 days	6-OHDA:<br>contra (f) approx. 1-2, (b) approx. 2-2.5; ipsi (f) approx. 13-15, (b) approx. 15-17<br>Sham: not done
Manfredsson et al. 2007 [36]	S-D, f	- SNpc<br>- 7 μg 6-OHDA<br>- not done	8 and 12 weeks	6-OHDA:<br>contra (f) approx. 2, (b) approx. 9; ipsi (f) approx. 12, (b) approx. 13<br>Sham: not done
Mendieta et al. 2012 [37]	W, m	- Left striatum<br>- 16 μg 6-OHDA<br>- not done	4 weeks	6-OHDA:<br>contra (f) 4.1 ± 0.4; ipsi (f) 13.5 ± 0.1<br>Sham:<br>contra (f) approx. 13
Nikkhah et al. 2001 [38]	S-D, f	- Left or right mesostriatal pathway<br>- 7.2 μg 6-OHDA<br>- not done	10 weeks	6-OHDA:<br>contra (f) approx. 1, (b) approx. 5-8; ipsi not shown<br>Sham: not done
Ostock et al. 2014 [39]	S-D, m	- Left MFB<br>- 12 μg 6-OHDA<br>- 25 mg/kg	3-4 weeks	6-OHDA: lesioned forehand steps/intact forehand steps (%)<br>contra (f) approx. 10%, (b) approx. 25%; ipsi not shown<br>Sham: not done
Rosenblad et al. 1998 [40]	S-D, f	- Right striatum<br>- 20 μg 6-OHDA<br>- not done	8 weeks	6-OHDA:<br>contra (f) 6.9 ± 1.0, (b) 10.9 ± 1.1; ipsi (f) 11.2 ± 0.8, (b) 14.1 ± 0.6<br>Sham: not done
Sampaio et al. 2017 [41]	W, m	- Right striatum<br>- 20 μg 6-OHDA<br>- not done	60 days	6-OHDA:<br>contra (f) approx. 4; ipsi (f) approx. 11<br>Sham:<br>contra (f) approx. 11, ipsi (f) approx. 11
Sander et al. 2012 [42]	S-D, f	- Left MFB<br>- 8 μg 6-OHDA<br>- not done	3 weeks	6-OHDA:<br>contra (f) approx. 5, ipsi not shown<br>Sham: not done
Seeger-Armbruster and Ameln-Mayerhofer 2013 [43]	S-D, m	- Left MFB<br>- 12 μg 6-OHDA<br>- 20 mg/kg	4 weeks	6-OHDA:<br>contra (f) approx. 3, (b) approx. 4; ipsi (f) approx. 18, (b) approx. 19<br>Sham:<br>contra (f) approx. 17, (b) approx. 17; ipsi (f) approx. 15, (b) approx. 17
Shin et al. 2014 [44]	S-D, f	- Right MFB<br>- 14 μg 6-OHDA	1 week	Contralateral paw touches rates of ipsilateral paw touches (%)<br>6-OHDA:

		- not done		contra (b) approx. 20%<br>Sham: not done
Sutton et al. 2013 [45]	S-D, m	- Right MFB<br>- 13.5 µg 6-OHDA<br>- 25 mg/kg desipramine + 50 mg/kg pargyline	3 weeks	6-OHDA :<br>Forelimb contact bias (% ipsilateral/total), (f) approx. 85%<br>Sham: not shown
Tronci et al. 2013 [46]	S-D, f	- MFB<br>- 16 µg<br>- not done	3 weeks	6-OHDA:<br>contra (f+b) approx. 2; ipsi not shown<br>Sham: not done
Tronci et al. 2014 [47]	S-D, m	- Right MFB<br>- 16 µg<br>- not done	3 weeks	6-OHDA:<br>contra (f+b) approx. 2; ipsi not shown<br>Sham: not done
Tronci et al. 2015 [48]	S-D, m	- MFB<br>- 16 µg<br>- not done	3 weeks	6-OHDA:<br>contra (f+b) approx. 2; ipsi not shown<br>Sham: not done
Winkler et al. 1996 [49]	S-D, f	- Right striatum<br>- 20 µg 6-OHDA<br>- not done	4 months	6-OHDA:<br>contra (f) approx. 3, (b) approx. 9; ipsi (f) approx. 11, (b) approx. 13<br>Sham: not done
Winkler et al. 2002 [50]	S-D, f	- Striatum<br>- 7 µg 6-OHDA<br>- MFB<br>- 10,5 µg 6-OHDA<br>- not done	3 weeks	6-OHDA: (Striatum and MFB)<br>contra (f) approx. 1-2; ipsi (f) approx. 10-12<br>Sham: not done
Yoon et al. 2014a [51]	W, m	- Right MFB<br>- 8 µg 6-OHDA<br>- not done	1, 4 and 6 weeks after optic fiber implantation	6-OHDA: rate of contra forelimb touches (%) in both forelimb touches<br>contra (b) approx. 15%<br>Sham: not done
Yoon et al. 2014b [52]	W, m	- Right MFB<br>- 8 µg 6-OHDA<br>- not done	2, 4, 8, and 9 weeks	6-OHDA: as contra paw touches rate (%) of the ipsi paw touches<br>contra (b) approx. 10-12%<br>Sham: not done

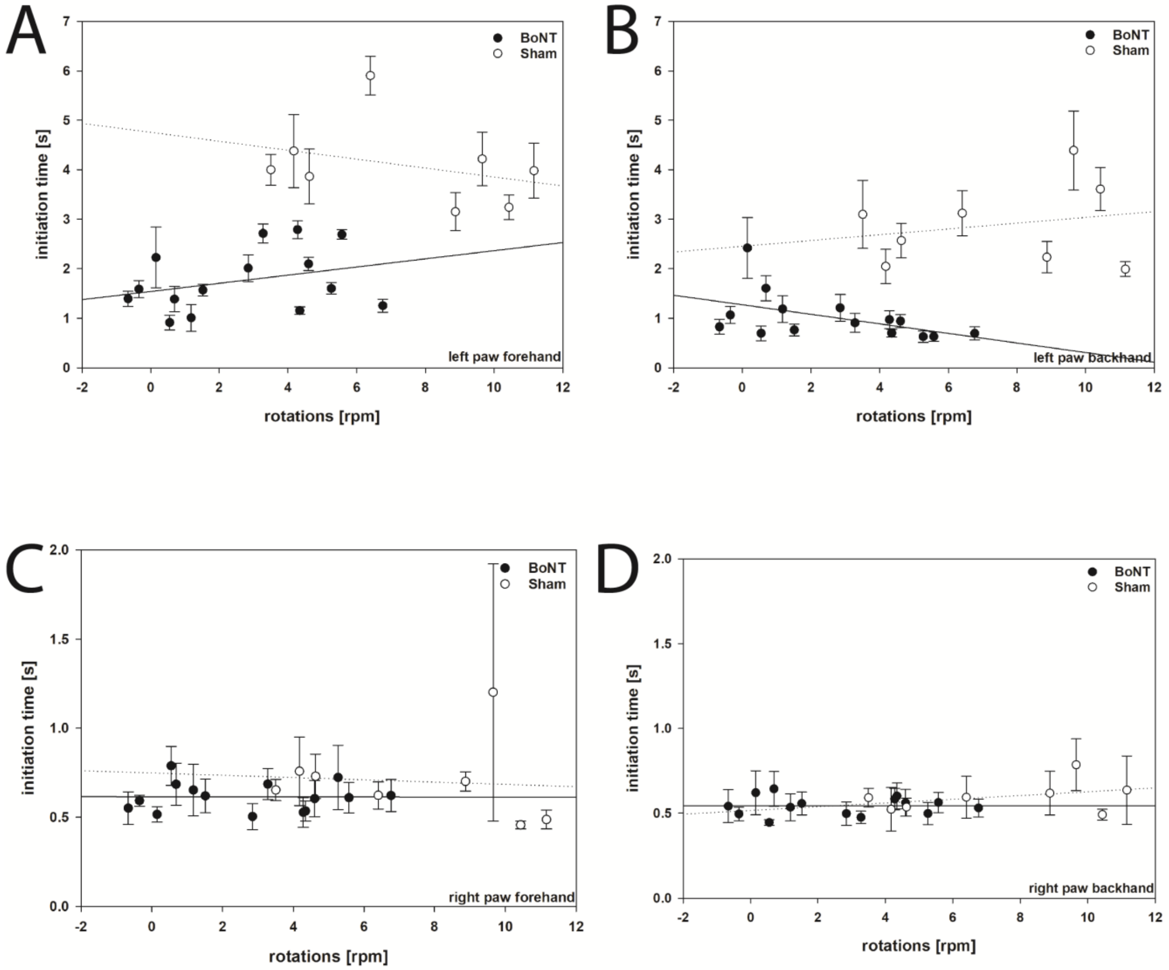

Figure S1. Correlation analysis of the initiation time of stepping movements of the left (contralateral) (A,B) and right (ipsilateral) (C,D) forelimbs in right side 6-OHDA-lesioned rats in both forehand (A,C) and backhand (B,D) directions 1 month after the 1. intrastriatal BoNT-A application and apomorphine-induced rotations. Data represent means ± SEM. Linear regression lines are displayed for BoNT (solid) and Sham (dotted) groups.

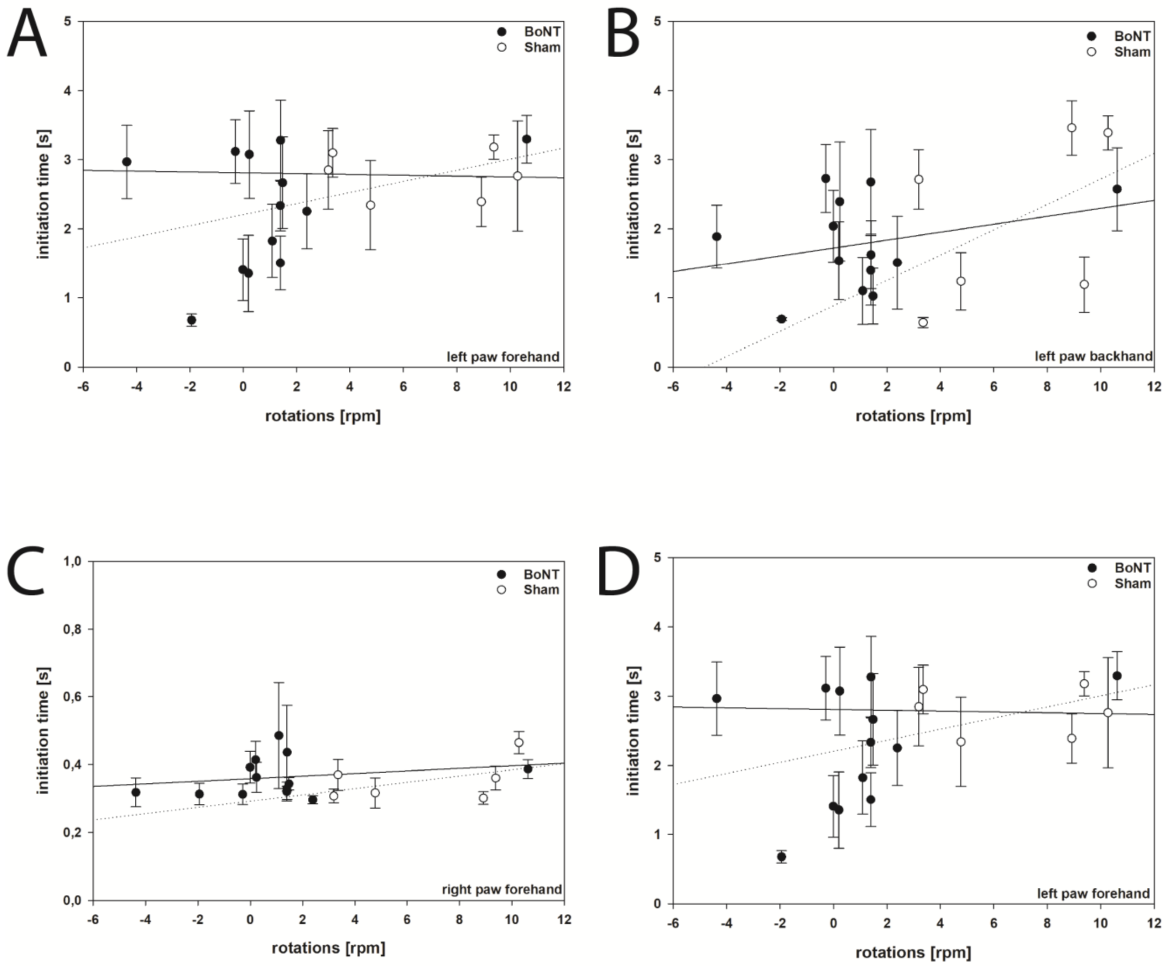

Figure S2. Correlation analysis of the initiation time of stepping movements of the left (contralateral) (A,B) and right (ipsilateral) (C,D) forelimbs in right side 6-OHDA-lesioned rats in both forehand (A,C) and backhand (B,D) directions 1 month after the 2. intrastriatal BoNT-A application and apomorphine-induced rotations. Data represent means ± SEM. Linear regression lines are displayed for BoNT (solid) and Sham (dotted) groups.

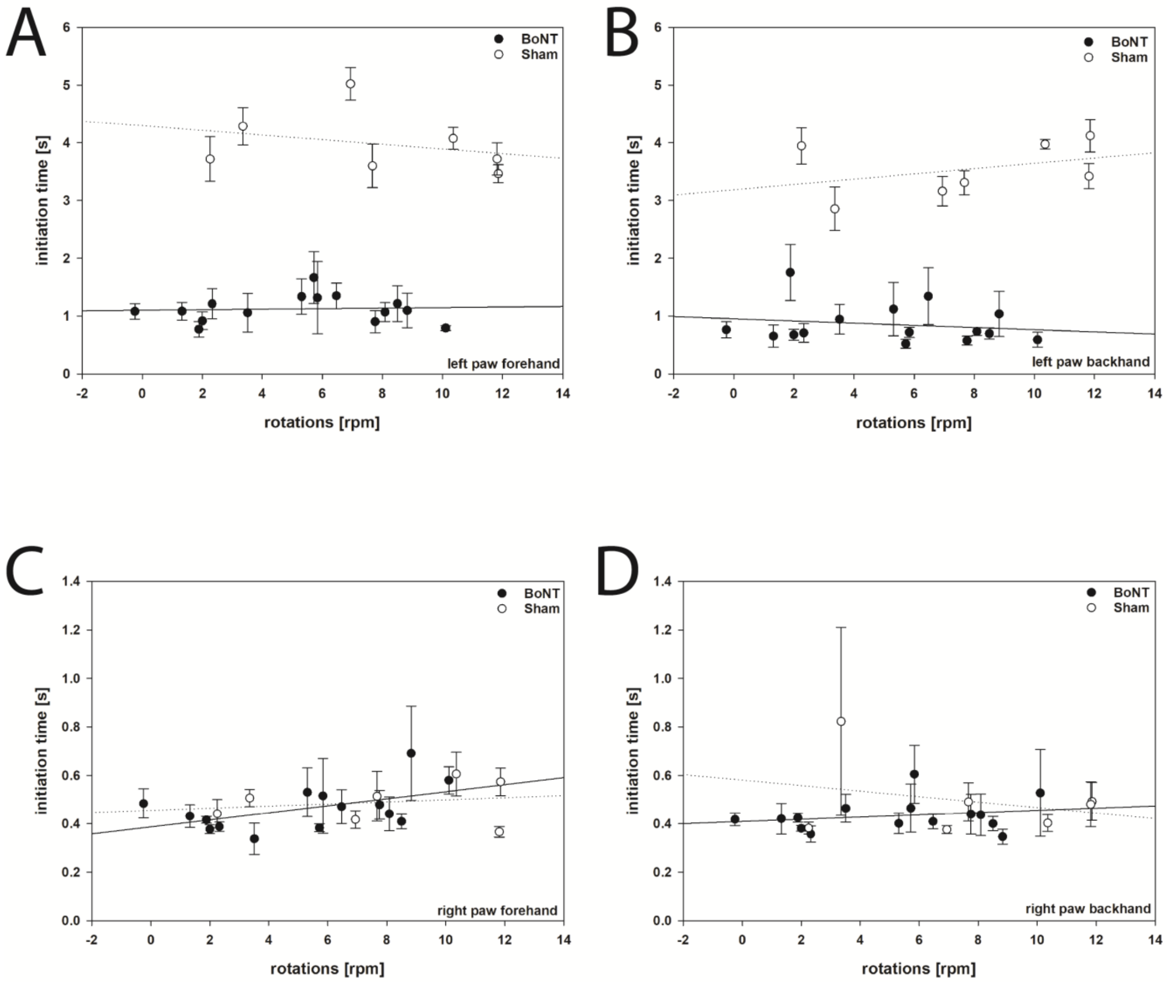

Figure S3. Correlation analysis of the initiation time of stepping movements of the left (contralateral) (A,B) and right (ipsilateral) (C,D) forelimbs in right side 6-OHDA-lesioned rats in both forehand (A,C) and backhand (B,D) directions 3 months after the 1. intrastriatal BoNT-A application and apomorphine-induced rotations. Data represent means ± SEM. Linear regression lines are displayed for BoNT (solid) and Sham (dotted) groups.

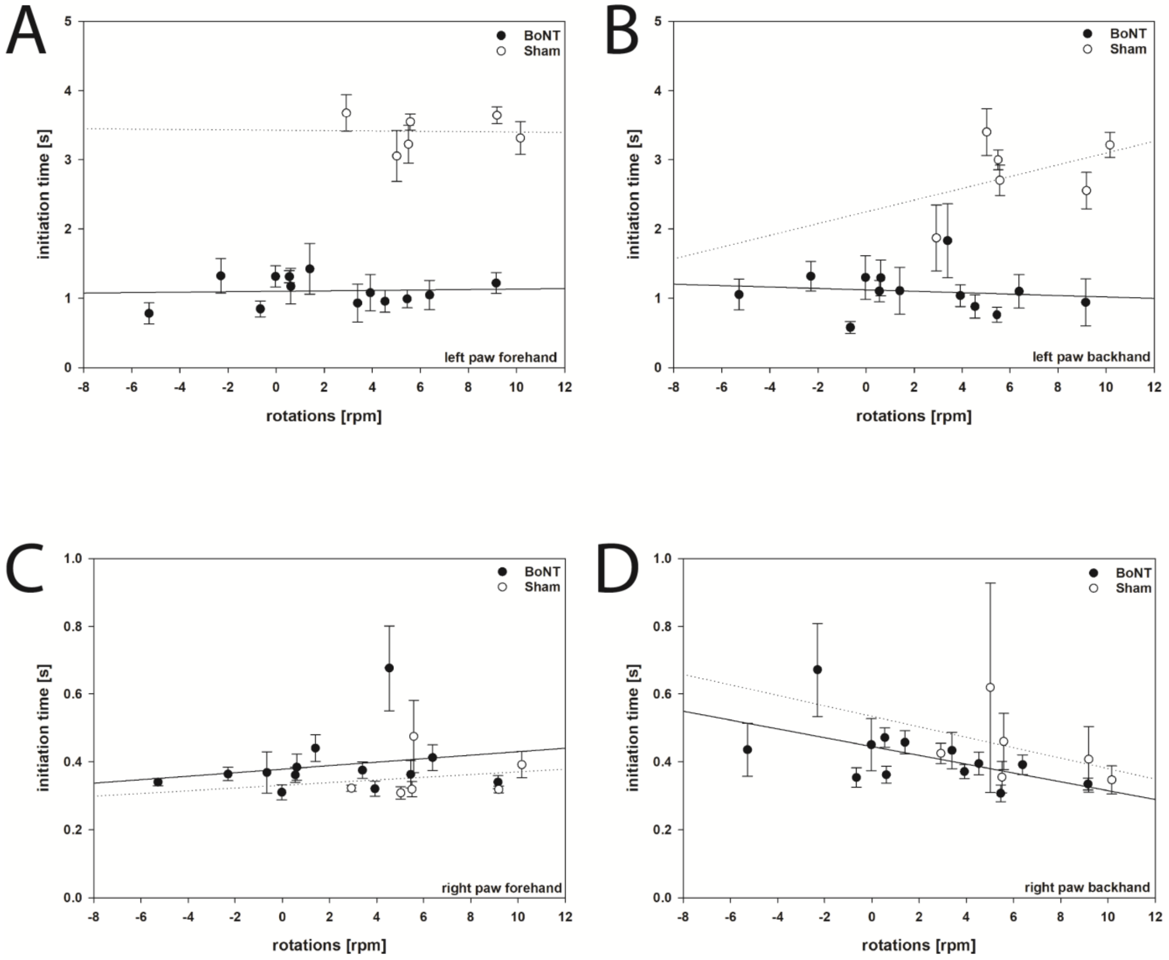

Figure S4. Correlation analysis of the initiation time of stepping movements of the left (contralateral) (A,B) and right (ipsilateral) (C,D) forelimbs in right side 6-OHDA-lesioned rats in both forehand (A,C) and backhand (B,D) directions 3 months after the 2. intrastriatal BoNT-A application and apomorphine-induced rotations. Data represent means ± SEM. Linear regression lines are displayed for BoNT (solid) and Sham (dotted) groups.

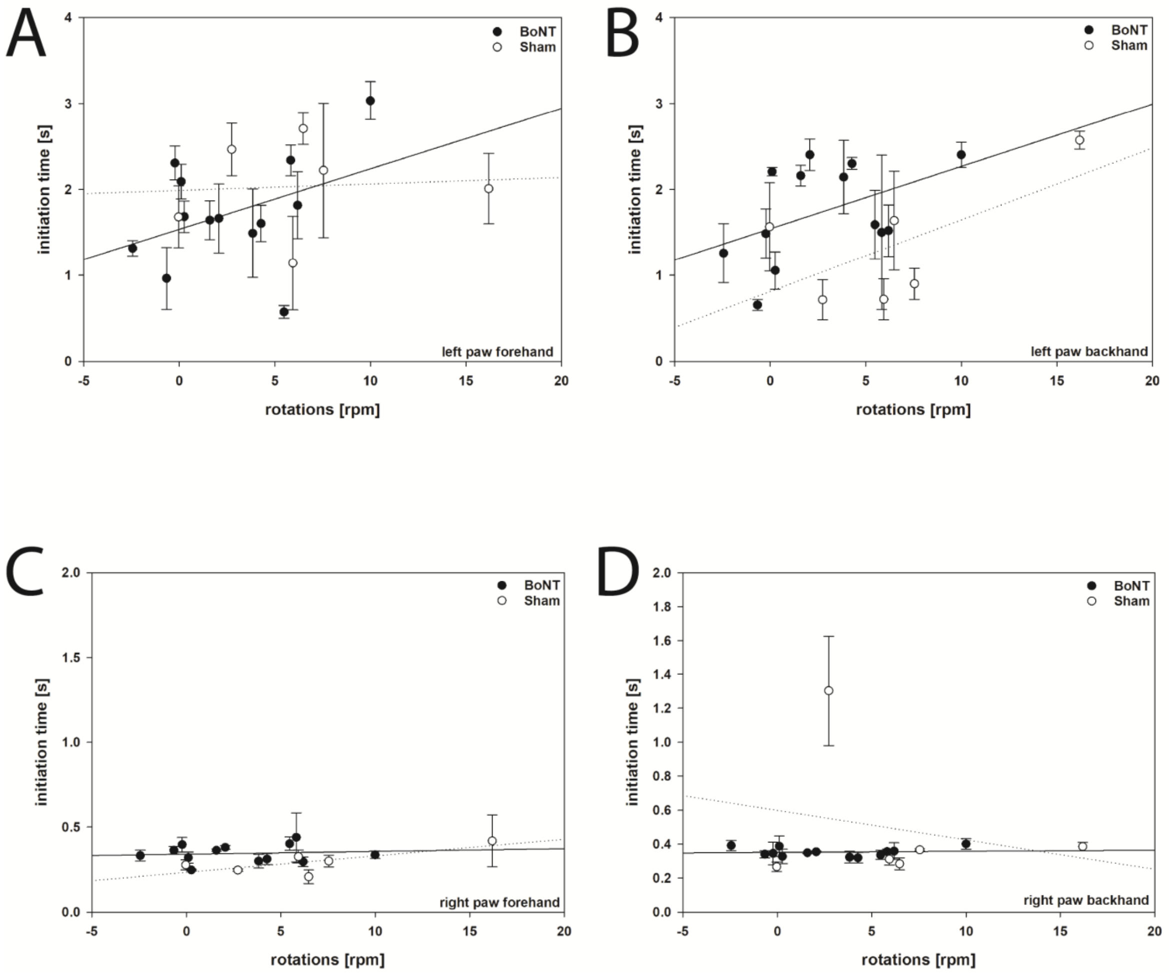

Figure S5. Correlation analysis of the initiation time of stepping movements of the left (contralateral) (A,B) and right (ipsilateral) (C,D) forelimbs in right side 6-OHDA-lesioned rats in both forehand (A,C) and backhand (B,D) directions 6 months after the 1. intrastriatal BoNT-A application and apomorphine-induced rotations. Data represent means ± SEM. Linear regression lines are displayed for BoNT (solid) and Sham (dotted) groups.

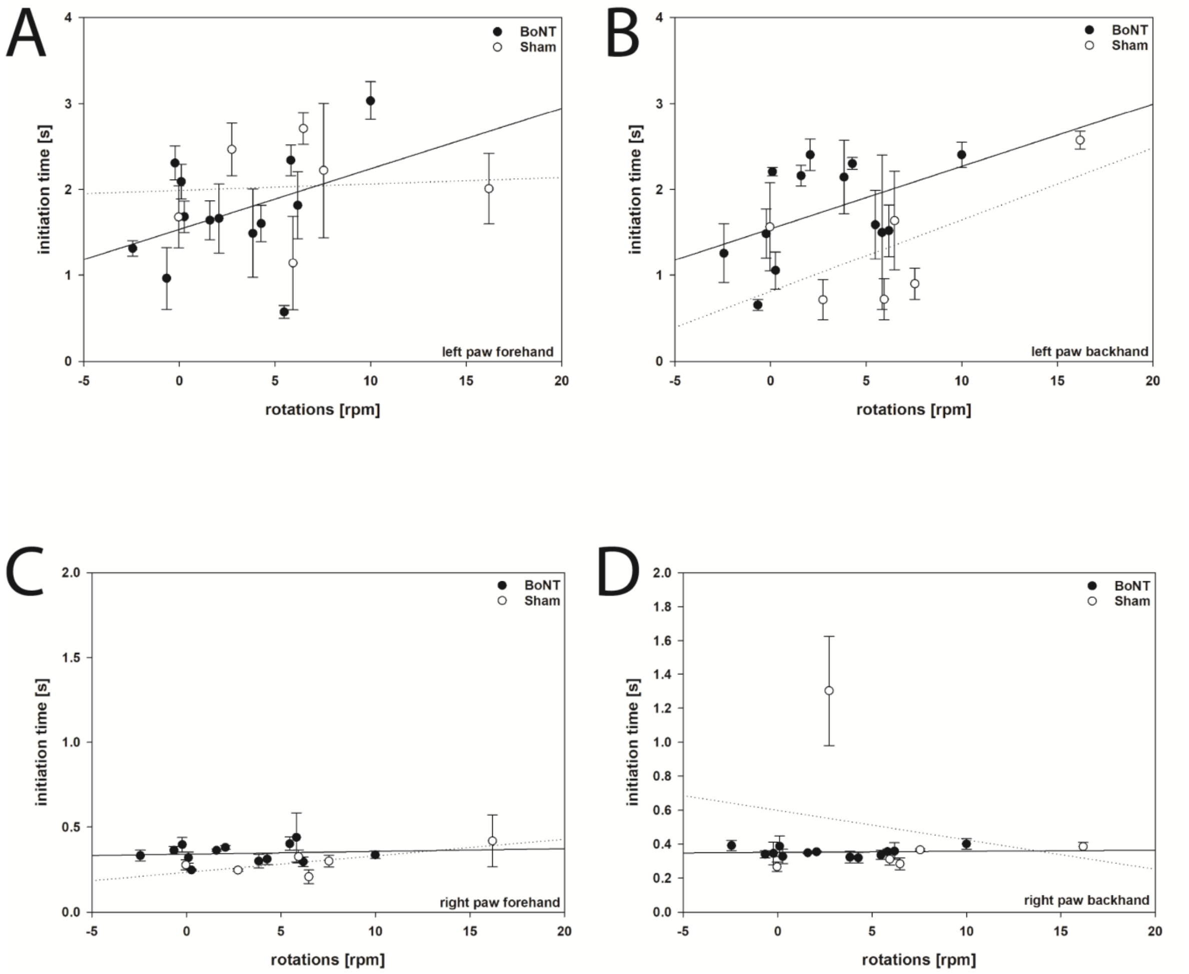

Figure S6. Correlation analysis of the initiation time of stepping movements of the left (contralateral) (A,B) and right (ipsilateral) (C,D) forelimbs in right side 6-OHDA-lesioned rats in both forehand (A,C) and backhand (B,D) directions 6 months after the 2. intrastriatal BoNT-A application and apomorphine-induced rotations. Data represent means ± SEM. Linear regression lines are displayed for BoNT (solid) and Sham (dotted) groups.

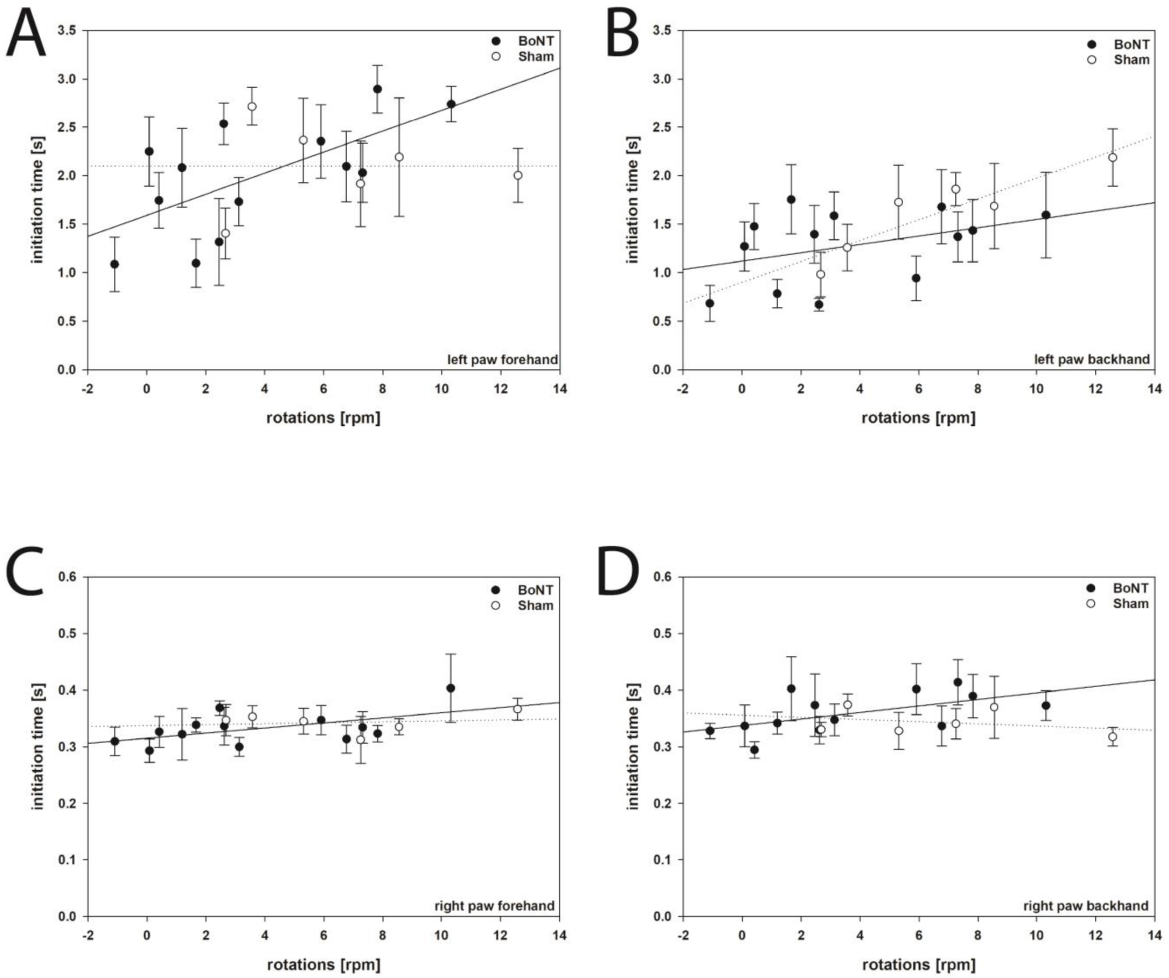

Figure S7. Correlation analysis of the initiation time of stepping movements of the left (contralateral) (A,B) and right (ipsilateral) (C,D) forelimbs in right side 6-OHDA-lesioned rats in both forehand (A,C) and backhand (B,D) directions 9 months after the 2. intrastriatal BoNT-A application and apomorphine-induced rotations. Data represent means ± SEM. Linear regression lines are displayed for BoNT (solid) and Sham (dotted) groups.

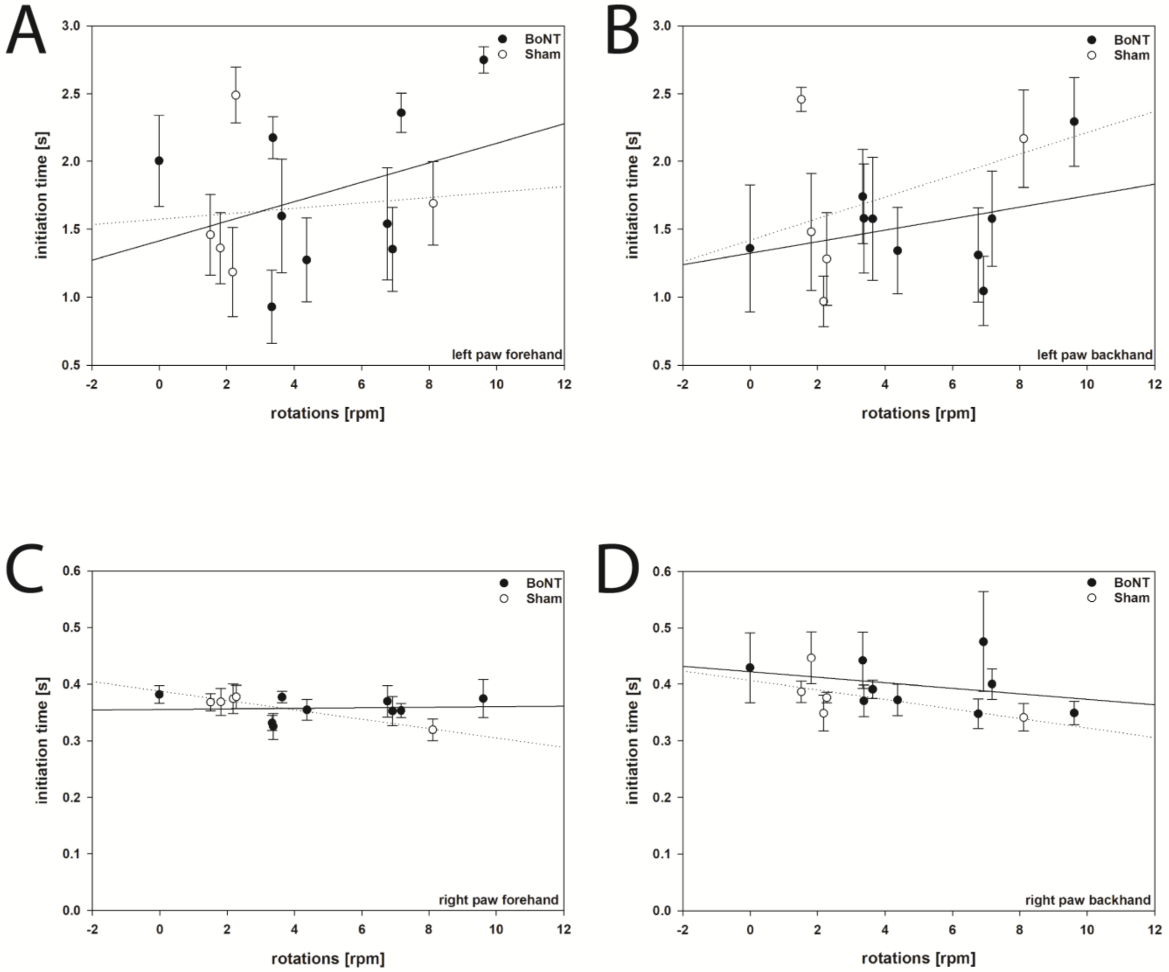

Figure S8. Correlation analysis of the initiation time of stepping movements of the left (contralateral) (A,B) and right (ipsilateral) (C,D) forelimbs in right side 6-OHDA-lesioned rats in both forehand (A,C) and backhand (B,D) directions 12 months after the 2. intrastriatal BoNT-A application and apomorphine-induced rotations. Data represent means ± SEM. Linear regression lines are displayed for BoNT (solid) and Sham (dotted) groups.

References

1. Antala, B.; Bhuva, S.; Gupta, S.; Lahkar, M.; Patel, M.; Rabadiya, S. Protective effect of methanolic extract of Garcinia indica fruits in 6-OHDA rat model of Parkinson's disease. *Indian J. Pharmacol.* **2012**, *44*, 683, doi:10.4103/0253-7613.103242.

2. Badstuebner, K.; Gimsa, U.; Weber, I.; Tuchscherer, A.; Gimsa, J. Deep Brain Stimulation of Hemiparkinsonian Rats with Unipolar and Bipolar Electrodes for up to 6 Weeks: Behavioral Testing of Freely Moving Animals. *Parkinsons. Dis.* **2017**, *2017*, 1–18, doi:10.1155/2017/5693589.

3. Fang, X.; Sugiyama, K.; Akamine, S.; Namba, H. Improvements in motor behavioral tests during deep brain stimulation of the subthalamic nucleus in rats with different degrees of unilateral parkinsonism. *Brain Res.* **2006**, *1120*, 202–210, doi:10.1016/j.brainres.2006.08.073.

4. Fang, X.; Sugiyama, K.; Akamine, S.; Namba, H. The stepping test and its learning process in different degrees of unilateral striatal lesions by 6-hydroxydopamine in rats. *Neurosci. Res.* **2006**, *55*, 403–9, doi:10.1016/j.neures.2006.04.010.

5. Fang, X.; Sugiyama, K.; Akamine, S.; Sun, W.; Namba, H. The different performance among motor tasks during the increasing current intensity of deep brain stimulation of the subthalamic nucleus in rats with different degrees of the unilateral striatal lesion. *Neurosci. Lett.* **2010**, *480*, 64–68, doi:10.1016/j.neulet.2010.06.004.
6. Mukhida, K.; Baker, K. A.; Sadi, D.; Mendez, I. Enhancement of sensorimotor behavioral recovery in hemiparkinsonian rats with intrastriatal, intranigral, and intrasubthalamic nucleus dopaminergic transplants. *J. Neurosci.* **2001**, *21*, 3521–3530, doi:21/10/3521 [pii].
7. Olsson, M.; Nikkhah, G.; Bentlage, C.; Björklund, A. Forelimb akinesia in the rat Parkinson model: differential effects of dopamine agonists and nigral transplants as assessed by a new stepping test. *J. Neurosci.* **1995**, *15*, 3863–3875.
8. Pinna, A.; Pontis, S.; Borsini, F.; Morelli, M. Adenosine A2A receptor antagonists improve deficits in initiation of movement and sensory motor integration in the unilateral 6-hydroxydopamine rat model of Parkinson's disease. *Synapse* **2007**, *61*, 606–614, doi:10.1002/syn.20410.
9. Pinna, A.; Tronci, E.; Schintu, N.; Simola, N.; Volpini, R.; Pontis, S.; Cristalli, G.; Morelli, M. A new ethyladenine antagonist of adenosine A2A receptors: Behavioral and biochemical characterization as an antiparkinsonian drug. *Neuropharmacology* **2010**, *58*, 613–623, doi:10.1016/j.neuropharm.2009.11.012.
10. Singh, S.; Ahmed, R.; Sagar, R. K.; Krishana, B. Neuroprotection of the nigrostriatal dopaminergic neurons by melatonin in hemiparkinsonium rat. *Indian J. Med. Res.* **2006**, *124*, 419–426.
11. Singh, S.; Ahmad, R.; Mathur, D.; Sagar, R. K.; Krishana, B.; Arora, R.; Sharma, R. K. Neuroprotective effect of BDNF in young and aged 6-OHDA treated rat model of Parkinson disease. *Indian J. Exp. Biol.* **2006**, *44*, 699–704.
12. Sun, W.; Sugiyama, K.; Fang, X.; Yamaguchi, H.; Akamine, S.; Magata, Y.; Namba, H. Different striatal D2-like receptor function in an early stage after unilateral striatal lesion and medial forebrain bundle lesion in rats. *Brain Res.* **2010**, *1317*, 227–235, doi:10.1016/j.brainres.2009.12.048.
13. Sun, W.; Sugiyama, K.; Asakawa, T.; Ito-Yamashita, T.; Namba, H. Behavioral performance at early (4 weeks) and later (6 months) stages in rats with unilateral medial forebrain bundle and striatal 6-hydroxydopamine lesions. *Neurol. Med. Chir. (Tokyo).* **2013**, *53*, 7–11, doi:10.2176/nmc.53.7.
14. Zhang, S.; Gui, X. H.; Huang, L. P.; Deng, M. Z.; Fang, R. M.; Ke, X. H.; He, Y. P.; Li, L.; Fang, Y. Q. Neuroprotective Effects of β-Asarone Against 6-Hydroxy Dopamine-Induced Parkinsonism via JNK/Bcl-2/Beclin-1 Pathway. *Mol. Neurobiol.* **2016**, *53*, 83–94, doi:10.1007/s12035-014-8950-z.
15. Abedi, P. M.; Delaville, C.; De Deurwaerdere, P.; Benjelloun, W.; Benazzouz, A. Intrapallidal administration of 6-hydroxydopamine mimics in large part the electrophysiological and behavioral consequences of major dopamine depletion in the rat. *Neuroscience* **2013**, *236*, 289–297, doi:10.1016/j.neuroscience.2013.01.043.

16. Acuña-Lizama, M. M.; Bata-García, J. L.; Alvarez-Cervera, F. J.; Góngora-Alfaro, J. L. Caffeine has greater potency and efficacy than theophylline to reverse the motor impairment caused by chronic but not acute interruption of striatal dopaminergic transmission in rats. *Neuropharmacology* **2013**, *70*, 51–62, doi:10.1016/j.neuropharm.2013.01.002.

17. Antipova, V.; Holzmann, C.; Schmitt, O.; Wree, A.; Hawlitschka, A. Botulinum Neurotoxin A Injected Ipsilaterally or Contralaterally into the Striatum in the Rat 6-OHDA Model of Unilateral Parkinson's Disease Differently Affects Behavior. *Front. Behav. Neurosci.* **2017**, *11*, 119, doi:10.3389/fnbeh.2017.00119.

18. Baker, K. A.; Sadi, D.; Hong, M.; Mendez, I. Simultaneous intrastriatal and intranigral dopaminergic grafts in the parkinsonian rat model: role of the intranigral graft. *J. Comp. Neurol.* **2000**, *426*, 106–16.

19. Barnéoud, P.; Descombris, E.; Aubin, N.; Abrous, D. N. Evaluation of simple and complex sensorimotor behaviours in rats with a partial lesion of the dopaminergic nigrostriatal system. *Eur. J. Neurosci.* **2000**, *12*, 322–336, doi:10.1046/j.1460-9568.2000.00896.x.

20. Betts, M. J.; O'Neill, M. J.; Duty, S. Allosteric modulation of the group III mGlu4 receptor provides functional neuroprotection in the 6-hydroxydopamine rat model of Parkinson's disease. *Br. J. Pharmacol.* **2012**, *166*, 2317–2330, doi:10.1111/j.1476-5381.2012.01943.x.

21. Bordia, T.; McGregor, M.; Papke, R. L.; Decker, M. W.; Michael McIntosh, J.; Quik, M. The α7 nicotinic receptor agonist ABT-107 protects against nigrostriatal damage in rats with unilateral 6-hydroxydopamine lesions. *Exp. Neurol.* **2015**, *263*, 277–284, doi:10.1016/j.expneurol.2014.09.015.

22. Cerri, S.; Greco, R.; Levandis, G.; Ghezzi, C.; Mangione, A. S.; Fuzzati-Armentero, M.-T.; Bonizzi, A.; Avanzini, M. A.; Maccario, R.; Blandini, F. Intracarotid Infusion of Mesenchymal Stem Cells in an Animal Model of Parkinson's Disease, Focusing on Cell Distribution and Neuroprotective and Behavioral Effects. *Stem Cells Transl. Med.* **2015**, *4*, 1073–1085, doi:10.5966/sctm.2015-0023.

23. Chotibut, T.; Meadows, S.; Kasanga, E. A.; McInnis, T.; Cantu, M. A.; Bishop, C.; Salvatore, M. F. Ceftriaxone reduces L-dopa–induced dyskinesia severity in 6-hydroxydopamine parkinson's disease model. *Mov. Disord.* **2017**, *32*, 1547–1556, doi:10.1002/mds.27077.

24. Dowd, E.; Monville, C.; Torres, E. M.; Wong, L.-F. F.; Azzouz, M.; Mazarakis, N. D.; Dunnett, S. B. Lentivector-mediated delivery of GDNF protects complex motor functions relevant to human Parkinsonism in a rat lesion model. *Eur. J. Neurosci.* **2005**, *22*, 2587–2595, doi:10.1111/j.1439-0442.1973.tb01060.x.

25. Dowd, E.; Dunnett, S. B. Comparison of 6-hydroxydopamine-induced medial forebrain bundle and nigrostriatal terminal lesions in a lateralised nose-poking task in rats. *Behav. Brain Res.* **2005**, *159*, 153–161, doi:10.1016/j.bbr.2004.10.010.

26. Frau, R.; Savoia, P.; Fanni, S.; Fiorentini, C.; Fidalgo, C.; Tronci, E.; Stancampiano, R.; Meloni, M.; Cannas, A.; Marrosu, F.; Bortolato, M.; Devoto, P.; Missale, C.; Carta, M. The 5-alpha reductase inhibitor finasteride reduces dyskinesia in a rat model of Parkinson's disease. *Exp. Neurol.* **2017**, *291*, 1–7, doi:10.1016/j.expneurol.2017.01.012.

27. Hahn, M.; Timmer, M.; Nikkhah, G. Survival and early functional integration of dopaminergic progenitor cells following transplantation in a rat model of Parkinson's disease. *J. Neurosci. Res.* **2009**, *87*, 2006–2019, doi:10.1002/jnr.22031.

28. Ivanova, E. A.; Kapitsa, I. G.; Val'dman, E. A.; Voronina, T. A. Anti-Parkinsonian Activity of Hemantane on a Model of Hemiparkinsonian Syndrome in Rats. *Bull. Exp. Biol. Med.* **2015**, *159*, 380–3, doi:10.1007/s10517-015-2968-8.

29. Kim, S. Y.; Choe, B. Y.; Lee, H. S.; Lee, D. W.; Ryu, K. N.; Park, J. S.; Yin, C. S.; Hong, K. S.; Lee, C. H.; Choi, C. B. Forelimb akinesia and metabolic alteration in the striatum following unilateral 6-hydroxydopamine lesion in rats: An in vivo proton magnetic resonance spectroscopy study. *Neurochem. J.* **2011**, *5*, 270–277, doi:10.1134/S1819712411040088.

30. Kirik, D.; Rosenblad, C.; Björklund, A. Characterization of behavioral and neurodegenerative changes following partial lesions of the nigrostriatal dopamine system induced by intrastriatal 6-hydroxydopamine in the rat. *Exp. Neurol.* **1998**, *152*, 259–77, doi:10.1006/exnr.1998.6848.

31. Kirik, D.; Rosenblad, C.; Bjorklund, A. Preservation of a functional nigrostriatal dopamine pathway by GDNF in the intrastriatal 6-OHDA lesion model depends on the site of administration of the trophic factor. *Eur. J. Neurosci.* **2000**, *12*, 3871–3882, doi:10.1046/j.1460-9568.2000.00274.x.

32. Kirik, D.; Winkler, C.; Björklund, A. Growth and functional efficacy of intrastriatal nigral transplants depend on the extent of nigrostriatal degeneration. *J. Neurosci.* **2001**, *21*, 2889–96, doi:21/8/2889 [pii].

33. Kirik, D.; Georgievska, B.; Rosenblad, C.; Björklund, A. Delayed infusion of GDNF promotes recovery of motor function in the partial lesion model of Parkinson's disease. *Eur. J. Neurosci.* **2001**, *13*, 1589–99.

34. Köllensperger, M.; Stefanova, N.; Reindl, M.; Poewe, W.; Wenning, G. K. Loss of dopaminergic responsiveness in a double lesion rat model of the Parkinson variant of multiple system atrophy. *Mov. Disord.* **2007**, *22*, 353–8, doi:10.1002/mds.21251.

35. Lettfuss, N. Y.; Fischer, K.; Sossi, V.; Pichler, B. J.; von Ameln-Mayerhofer, A. Imaging DA release in a rat model of L-DOPA-induced dyskinesias: A longitudinal in vivo PET investigation of the antidyskinetic effect of MDMA. *Neuroimage* **2012**, *63*, 423–433, doi:10.1016/j.neuroimage.2012.06.051.

36. Manfredsson, F. P.; Burger, C.; Sullivan, L. F.; Muzyczka, N.; Lewin, A. S.; Mandel, R. J. rAAV-mediated nigral human parkin over-expression partially ameliorates motor deficits via enhanced dopamine neurotransmission in a rat model of Parkinson's disease. *Exp. Neurol.* **2007**, *207*, 289–301, doi:10.1016/j.expneurol.2007.06.019.

37. Mendieta, L.; Bautista, E.; Sánchez, A.; Guevara, J.; Herrando-Grabulosa, M.; Moran, J.; Martínez, R.; Aguilera, J.; Limón, I. D. The C-terminal domain of the heavy chain of tetanus toxin given by intramuscular injection causes neuroprotection and improves the motor behavior in rats treated with 6-hydroxydopamine. *Neurosci. Res.* **2012**, *74*, 156–167, doi:10.1016/j.neures.2012.08.006.

38. Nikkhah, G.; Falkenstein, G.; Rosenthal, C. Restorative plasticity of dopamine neuronal transplants depends on the degree of hemispheric dominance. *J. Neurosci.* **2001**, *21*, 6252–63.

39. Ostock, C. Y.; Lindenbach, D.; Goldenberg, A. A.; Kampton, E.; Bishop, C. Effects of noradrenergic denervation by anti-DBH-saporin on behavioral responsivity to l-DOPA in the hemi-parkinsonian rat. *Behav. Brain Res.* **2014**, *270*, 75–85, doi:10.1016/j.bbr.2014.05.009.

40. Rosenblad, C.; Martinez-Serrano, A.; Björklund, A. Intrastriatal glial cell line-derived neurotrophic factor promotes sprouting of spared nigrostriatal dopaminergic afferents and induces recovery of function in a rat model of Parkinson's disease. *Neuroscience* **1998**, *82*, 129–37.

41. Sampaio, T. B.; Pinton, S.; da Rocha, J. T.; Gai, B. M.; Nogueira, C. W. Involvement of BDNF/TrkB signaling in the effect of diphenyl diselenide on motor function in a Parkinson's disease rat model. *Eur. J. Pharmacol.* **2017**, *795*, 28–35, doi:10.1016/j.ejphar.2016.11.054.

42. Sander, S. E.; Lemm, C.; Lange, N.; Hamann, M.; Richter, A. Retigabine, a K V7 (KCNQ) potassium channel opener, attenuates l-DOPA-induced dyskinesias in 6-OHDA-lesioned rats. *Neuropharmacology* **2012**, *62*, 1052–1061, doi:10.1016/j.neuropharm.2011.10.016.

43. Seeger-Armbruster, S.; Von Ameln-Mayerhofer, A. Short- and long-term unilateral 6-hydroxydopamine lesions in rats show different changes in characteristics of spontaneous firing of substantia nigra pars reticulata neurons. *Exp. Brain Res.* **2013**, *224*, 15–24, doi:10.1007/s00221-012-3285-3.

44. Shin, E. S.; Hwang, O.; Hwang, Y. S.; Suh, J. K. F.; Chun, Y. Il; Jeon, S. R. Enhanced efficacy of human brain-derived neural stem cells by transplantation of cell aggregates in a rat model of parkinson's disease. *J. Korean Neurosurg. Soc.* **2014**, *56*, 383–389, doi:10.3340/jkns.2014.56.5.383.

45. Sutton, A. C.; Yu, W.; Calos, M. E.; Mueller, L. E.; Berk, M.; Shim, J.; Molho, E. S.; Brotchie, J. M.; Carlen, P. L.; Shin, D. S. Elevated potassium provides an ionic mechanism for deep brain stimulation in the hemiparkinsonian rat. *Eur. J. Neurosci.* **2013**, *37*, 231–41, doi:10.1111/ejn.12040.

46. Tronci, E.; Lisci, C.; Stancampiano, R.; Fidalgo, C.; Collu, M.; Devoto, P.; Carta, M. 5-Hydroxy-tryptophan for the treatment of l-DOPA-induced dyskinesia in the rat Parkinson's disease model. *Neurobiol. Dis.* **2013**, *60*, 108–114, doi:10.1016/j.nbd.2013.08.014.

47. Tronci, E.; Fidalgo, C.; Zianni, E.; Collu, M.; Stancampiano, R.; Morelli, M.; Gardoni, F.; Carta, M. Effect of memantine on L-DOPA-induced dyskinesia in the 6-OHDA-lesioned rat model of Parkinson's disease. *Neuroscience* **2014**, *265*, 245–52, doi:10.1016/j.neuroscience.2014.01.042.

48. Tronci, E.; Fidalgo, C.; Stancampiano, R.; Carta, M. Effect of selective and non-selective serotonin receptor activation on l-DOPA-induced therapeutic efficacy and dyskinesia in parkinsonian rats. *Behav. Brain Res.* **2015**, *292*, 300–304, doi:10.1016/j.bbr.2015.06.034.

49. Winkler, C.; Sauer, H.; Lee, C. S.; Bjorklund, A. Short-Term GDNF Treatment Provides Long-Term Rescue of Lesioned Nigral Dopaminergic Neurons in a Rat Model of Parkinson ′ s Disease. *J. Neurosci.* **1996**, *16*, 7206–7215.

50. Winkler, C.; Kirik, D.; Björklund, A.; Cenci, M. A. L-DOPA-induced dyskinesia in the

intrastriatal 6-hydroxydopamine model of parkinson's disease: relation to motor and cellular parameters of nigrostriatal function. *Neurobiol. Dis.* **2002**, *10*, 165–186, doi:10.1006/nbdi.2002.0499.

51. Yoon, H. H.; Park, J. H.; Kim, Y. H.; Min, J.; Hwang, E.; Lee, C. J.; Francis Suh, J.-K.; Hwang, O.; Jeon, S. R.; Suh, J.-K. F.; Hwang, O.; Jeon, S. R. Optogenetic inactivation of the subthalamic nucleus improves forelimb akinesia in a rat model of Parkinson disease. *Neurosurgery* **2014**, *74*, 533–540, doi:10.1227/NEU.0000000000000297.

52. Yoon, H. H.; Kim, Y. H.; Shin, E. S.; Jeon, S. R. A rat model of striatonigral degeneration generated by simultaneous injection of 6-hydroxydopamine into the medial forebrain bundle and quinolinic acid into the striatum. *J. Korean Med. Sci.* **2014**, *29*, 1555–61, doi:10.3346/jkms.2014.29.11.1555.

© 2018 by the authors. Submitted for possible open access publication under the terms and conditions of the Creative Commons Attribution (CC BY) license (http://creativecommons.org/licenses/by/4.0/).
